# Description of a new genus and three new species of Otothyrinae (Siluriformes, Loricariidae)

**DOI:** 10.3897/zookeys.534.6169

**Published:** 2015-11-11

**Authors:** Fábio F. Roxo, Gabriel S. C. Silva, Luz E. Ochoa, Claudio Oliveira

**Affiliations:** 1 Universidade Estadual Paulista, Departamento de Morfologia, Laboratório de Biologia e Genética de Peixes, Rubião Júnior s/n, 18618–970 Botucatu, São Paulo State, Brazil

**Keywords:** Cascudinhos, freshwater fishes, systematic, *Hisonotus*, taxonomy

## Abstract

The genus *Hisonotus* was resurrected as a member of the tribe Otothyrini (actually subfamily Otothyrinae). However, phylogenetic studies based on morphological and molecular data showed that *Hisonotus* is not monophyletic and independent lineages can be identified, such as the group composed of the species *Hisonotus insperatus*, *Hisonotus luteofrenatus*, *Hisonotus oliveirai*, *Hisonotus paresi* and *Hisonotus piracanjuba*, a lineage unrelated to that containing the type species of the genus *Hisonotus* (*Hisonotus notatus*). Herein, based in molecular and morphological data, a new genus is described to accommodate the lineage mentioned above, into which are also added three new species. This new genus can be distinguished from other genera of Otothyrinae by the following combination of characters: (1) a pair of rostral plates at the tip of the snout; (2) two large pre-nasal plates just posterior to the rostral plates; (3) a supra-opercular plate that receives the laterosensory canal from the compound pterotic before the preopercle; (4) a well developed membrane at anal opening in females; and (5) a V-shaped spinelet. A key to species of *Curculionichthys* is provided.

## Introduction

The subfamily Otothyrinae (*sensu* Chiachio et al. 2008 and Roxo et al. 2014a) is one of the most diverse members of Loricariidae, distributed through almost all South America, in hydrographic systems from the Amazon to northern Argentina. Within this subfamily, the genus *Hisonotus* Eigenmann & Eigenmann, 1889 is composed of 35 species (Eschmeyer 2015) in drainages of southern and southeastern Brazil, from the Rio Uruguay basin, upper Rio Paraná, Laguna dos Patos and Coastal drainages extending from Rio Grande do Sul State to Rio de Janeiro State and the Amazon basin. This genus was resurrected by Schaefer (1998a) with the combination of the following characters: reduced or absent snout plates in the anterior portion of the nostril, rostrum with enlarged odontodes, and thickened plates forming the lateral rostral margin. However, Britski and Garavello (2007) argued that the characters used by Schaefer (1998a) for the definition of *Hisonotus*, as well as other genera of the Otothyrinae, needed to be redefined. For example, Britski and Garavello (2007) observed that a rostrum with enlarged odontodes is present in several genera and species of Otothyrinae, as well as in *Parotocinclus* Eigenmann & Eigenmann, 1889. Furthermore, Britski and Garavello (2007) suggested that the other two characters were also unsatisfactory to define *Hisonotus*.

Several molecular (e.g. Chiachio et al. 2008; Cramer et al. 2011; Roxo et al. 2014a) and morphological (e.g. Martins et al. 2014) studies suggested that *Hisonotus* was polyphyletic, with *Hisonotus insperatus* Britski & Garavello, 2003, *Hisonotus luteofrenatus* Britski & Garavello, 2007, *Hisonotus oliveirai* Roxo, Zawadzki & Troy, 2014b, *Hisonotus paresi* Roxo, Zawadzki & Troy, 2014b and *Hisonotus piracanjuba* Martins & Langeani, 2012 belonging to a lineage unrelated to the one that includes the type species, *Hisonotus notatus* Eigenmann & Eigenmann, 1889. In this way, the elucidation of the relationships of the members of the *Hisonotus* is important to understand the evolution of Otothyrinae as a whole, considering that this genus represents about 35% of the diversity of this subfamily. Herein, a new genus is proposed to accommodate the above-cited species of *Hisonotus* and three additional new species are described in this new genus.

## Material and methods

Measurements and counts were taken from the left side of the fish, and were made from point to point to the nearest 0.01 mm with a digital caliper. Body plate and osteology nomenclature follows Schaefer (1997) and measurements follow Carvalho and Reis (2009), except for body depth at dorsal fin origin. Abbreviations used in the text followed Carvalho and Reis (2009). Morphometrics are given as percentages of standard length (SL), except for subunits of the head region that are expressed as percentages of head length (HL). Specimens were cleared and double stained (c&s) according to the method of Taylor and Van Dyke (1985). Vertebral counts also include the five vertebrae that comprise the Weberian apparatus and the compound caudal centrum (PU1 + U1) as one element. Dorsal fin ray counts include the spinelet as the first unbranched ray. Institutional acronyms follow Fricke and Eschmeyer (2015). Specimens are deposited at the LBP, Laboratório de Biologia e Genética de Peixes, Universidade Estadual Paulista, Botucatu; MZUSP, Museu de Zoologia, Universidade de São Paulo, São Paulo; NUP, Coleção Ictiológica do Nupelia, Universidade Estadual de Maringá, Maringá. Zoological nomenclature follows the International Code of Zoological Nomenclature (International Commission on Zoological Nomenclature 1999).

## Results

### Description of the new genus *Curculionichthys*

#### Curculionichthys gen. n.

Taxon classificationAnimaliaSiluriformesLoricariidae

http://zoobank.org/B074B13E-26CB-41FB-B319-FBF81A58F6DC

##### Type species.

*Curculionichthys insperatus* (Britski & Garavello, 2003), new combination.

##### Diagnosis.

The new genus can be distinguished from all other Otothyrinae species by the following combination of characters: (1) a pair of rostral plates at the tip of the snout; (2) the presence of two large pre-nasal plates just posterior to the rostral plates; (3) a supra-opercular plate that receives the laterosensory canal from the compound pterotic before the preopercle; (4) a well developed membrane at anal opening in females; and (5) the presence of a V-shaped spinelet.

##### Etymology.

*Curculionichthys*, from the Latin “curculionem” (elongated snout) and from the Greek “ichthys” (fishes) related to the relatively elongated snouts of the fish species included in this genus.

##### Discussion of the new genus.

Schaefer (1998a) resurrected *Hisonotus* using characters that were considered ambiguous by Britski and Garavello (2007) and needed to be redefined. The hypothesis of monophyly of *Hisonotus* was rejected by Roxo et al. (2014a) and Martins et al. (2014). Roxo et al. (2014a) found *Hisonotus acuen* (*Hisonotus* sp. 3, Fig. 3 in Roxo et al. 2014a) closely related to *Hisonotus chromodontus*, *Parotocinclus* sp. 3 and *Parotocinclus aripuanensis*. The species *Hisonotus vespuccii* (*Hisonotus* sp. 1, Fig. 3 in Roxo et al. 2014a) appeared closely related to Parotocinclus aff. spilurus and a new species of Otothyrinae (*Hisonotus* sp. 2 from municipality of Jaíba, Minas Gerais State in Rio São Francisco basin). The species *Hisonotus bocaiuva* appeared closely related to species of *Parotocinclus* from Rio São Francisco (i.e. *Parotocinclus prata* and *Parotocinclus robustus*, Fig. 4 in Roxo et al. 2014a), *Parotocinclus bahiensis* and two new taxa (New taxon 1 and New taxon 2).

**Figure 1.**
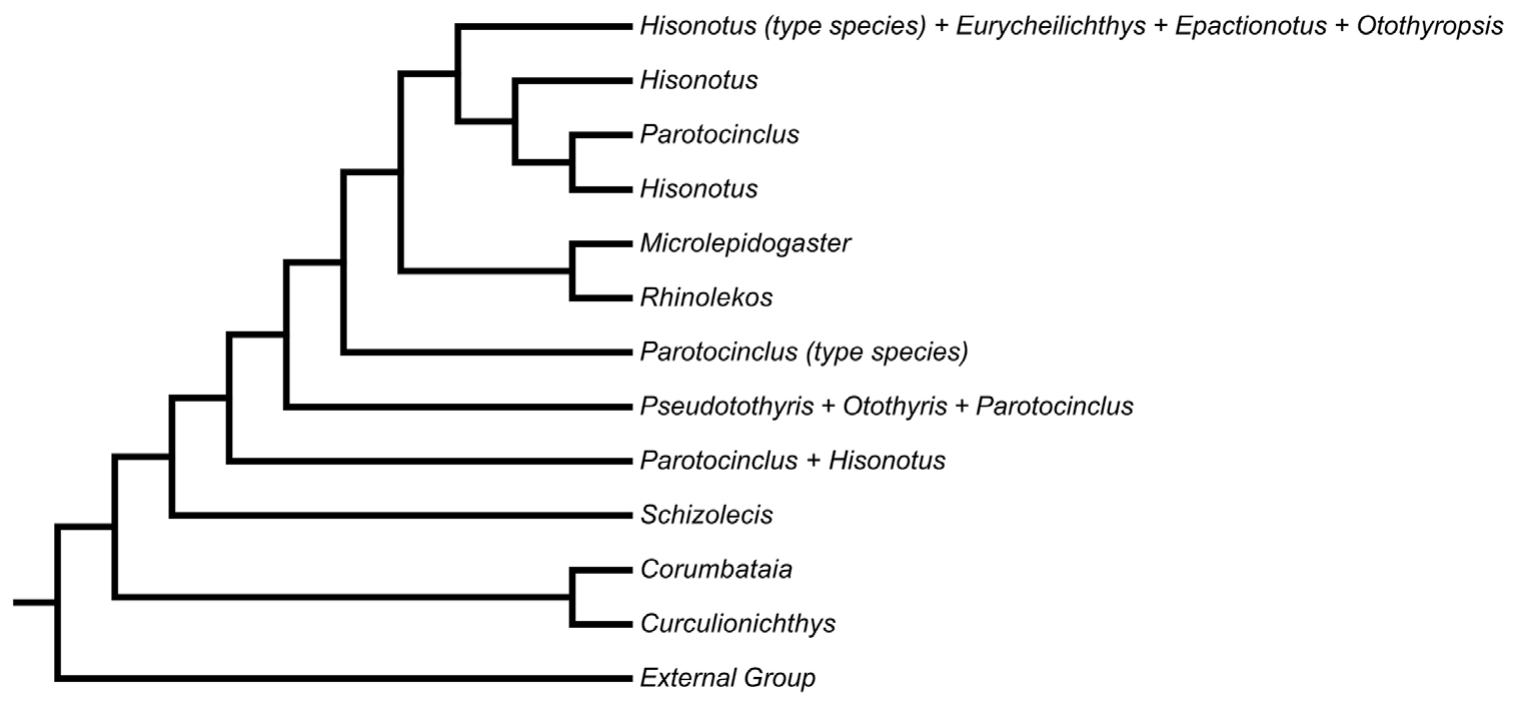
Dendrogram showing the phylogenetic relationship among Otothyrinae genera from the work of Roxo et al. (2014a). This figure shows the position of *Curculionichthys* close related with the genus *Corumbataia* and not related with the type species of the genus *Hisonotus* (i.e. *Hisonotus notatus*).

On the other hand, the species *Curculionichthys insperatus*, *Curculionichthys luteofrenatus*, *Curculionichthys oliveirai*, *Curculionichthys paresi* and *Curculionichthys piracanjuba* form a monophyletic group that is unrelated with the type species *Hisonotus notatus*, but instead with species of *Corumbataia* in Roxo et al. (2014a – using molecular data) and with *Hypoptopoma inexspectatum*, *Niobichthys ferrarisi*, *Otocinclus affinis*, *Oxyropsis acutirostra* and *Acestridium martini* in Martins et al. (2014 - using morphological data) (see Fig. 1 in the present paper for illustration of the phylogenetic position of *Curculionichthys* with the subfamily Otothyrinae according to Roxo et al. 2014a). In the present study, based in the information published in Roxo et al. (2014a) and in new morphological analyses, we propose the new genus, *Curculionichthys*, for re-allocation of five species described within *Hisonotus*: *Curculionichthys insperatus*, *Curculionichthys luteofrenatus*, *Curculionichthys oliveirai*, *Curculionichthys paresi* and *Curculionichthys piracanjuba* (see Table 2) and include three new species: *Curculionichthys sabaji*, *Curculionichthys coxipone*, and *Curculionichthys sagarana*. Four putative additional species are recognized in the analyzed material, but these species cannot be described yet due to the lack of sufficient specimens.

**Figure 2.**
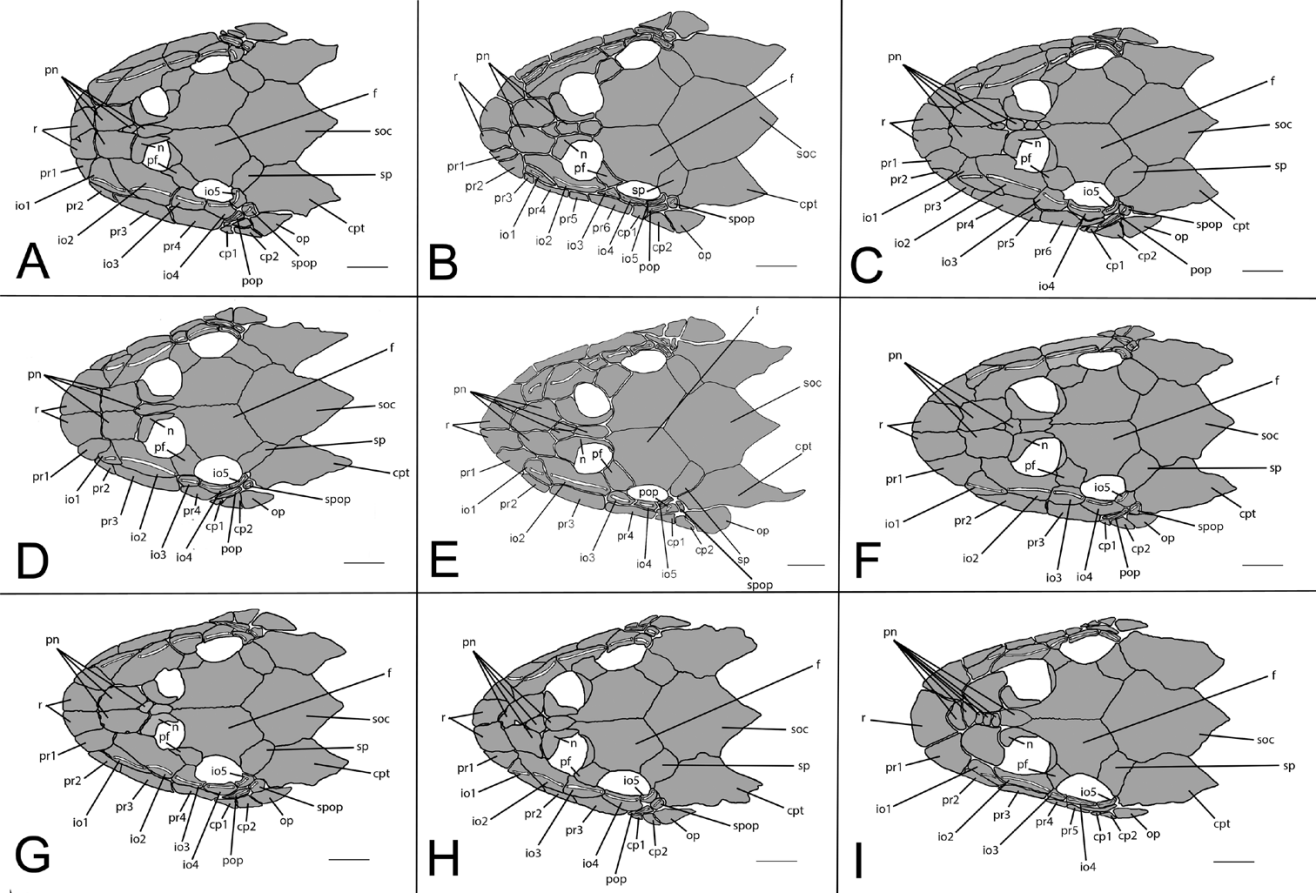
Cranial bones plates of the head in dorsal view of *Curculionichthys* species and the type species of the genus *Hisonotus*. **A** *Curculionichthys coxipone* **B** *Curculionichthys insperatus* **C** *Curculionichthys luteofrenatus* **D** *Curculionichthys oliveirai* **E** *Curculionichthys paresi* **F** *Curculionichthys piracanjuba* **G** *Curculionichthys sabaji* **H** *Curculionichthys sagarana* **I** *Hisonotus notatus*. Scale bar: 1 mm.

The new genus *Curculionichthys* is defined by the following combination of characters: (1) a pair of rostral plates at the tip of the snout; (2) the presence of two large pre-nasal plates just posterior to the rostral plates; (3) a supra-opercular plate that receives the laterosensory canal from the compound pterotic before the preopercle; (4) a well developed sexual dimorphic membrane at anal opening in females; and (5) the presence of a V-shaped spinelet. The tip of the snout that is composed of a pair of rostral plates (Fig. 2) was first reported in species of *Hisonotus* by Britski and Garavello (2003) in the description of *Hisonotus insperatus* (*Curculionichthys insperatus*), the type species of the new genus *Curculionichthys*. This character state according to Martins and Langeani (2012) is shared with *Corumbataia cuestae* Britski, 1997, species of *Microlepidogaster* Eigenmann & Eigenmann, 1889 (except *Microlepidogaster longicolla* Calegari & Reis, 2010), *Otothyris* Myers, 1927, and in all genera of Hypoptopomatinae (except in *Hypoptopoma* Gunther, 1868). We also observed that *Rhinolekos capetinga*, a species recently described from the Rio Tocantins basin, also have a pair of rostral plates. However, the morphology of this character in the species of *Curculionichthys* is different, as described by Martins and Langeani (2012), since the rostral plates are very large, the length of each plate is greater than their width and are more conspicuous when compared with all species listed previously in which the pair of rostral plates is smaller and have a quadrangular form.

The second character used to diagnose the new genus is the presence of two large pre-nasal plates just posterior to the rostral plates (Fig. 2). The pre-nasal plates present some variation in members of Otothyrinae, with respect to their numbers and shapes. In most species of Otothyrinae the pre-nasal plates are small or very tiny, however in species of *Curculionichthys* we found two very large pre-nasal plates just posterior to the rostral plates. However, even in species of *Curculionichthys* we can find variation in pre-nasal plates contacting the frontal and the nasal plates, but the two large pre-nasal plates just posterior to the rostral plate apparently is a synapomorphic character exclusive to *Curculionichthys*.

The presence of a supra-opercular plate that receives the laterosensory canal from the compound pterotic before the preopercle is the third character used to diagnose the new genus. According to Martins and Langeani (2012) this character is present in a large number of species of Loricariidae, but absent in the Hypoptopomatinae and Otothyrinae, except in the new genus *Curculionichthys*. The fourth character is the presence of a well developed membrane at anal opening in females. Both sex of *Curculionichthys* species have a membrane on the anal opening, however, it is more developed in females than in males, covering almost the entire urogenital opening. This character was first reported by Roxo et al. (2014b) in the description of *Curculionichthys oliveirai* and *Curculionichthys paresi* and it is absent in all other species of Otothyrinae, in which the membrane at anal opening is poorly developed (see Fig. 4 in Roxo et al. 2014b for illustration about this character states).

The fifth character used to diagnose *Curculionichthys* was the presence of a V-shaped spinelet in the dorsal fin. This character was first reported by Carvalho and Datovo (2012) in the description of *Hisonotus bockmanni* in personal communication with Roberto E. Reis. This character is not exclusive to *Curculionichthys* and it is shared with *Hisonotus acuen*, *Hisonotus chromodontus*, *Hisonotus vespuccii* and two new species of *Parotocinclus*, one from Xingu basin (LBP 15894) and the other one from Barra do Garça (LBP 12274). Furthermore, the V-shaped spinelet is shared with vast majority of Hypostominae species (Silva et al. 2014). However, within Otothyrinae it is good character that distinguishes the new genus.

In the description of *Curculionichthys oliveirai* and *Curculionichthys paresi*, Roxo et al. (2014b) found variation in head plate shape and number in the last two species and in *Curculionichthys insperatus*, even though osteological characters are generally conserved within Otothyrinae and Hypoptopomatinae (Schaefer 1987, 1997, 1998b; Garavello 1977; Mo 1991; de Pinna 1998; Diogo et al. 2001; Ribeiro et al. 2005). Roxo et al. (2014b) analyzed 18 specimens of *Curculionichthys insperatus* from type localities in Rio Capivara and Rio Araquá, from Botucatu, São Paulo State, three individuals presented a single rostral plate, instead of a pair of rostral plates (see Fig. 8 in Roxo et al. 2014b for variation of all characters). In *Curculionichthys oliveirai* and *Curculionichthys insperatus* the authors found bilateral asymmetry in the first infraorbital and the first and second posterior rostral plates and in an extra plate is found between preopercle and compound pterotic (known in the present study as our third character: a supra-opercular plate that receives the laterosensory canal from the compound pterotic before the preopercle). Despite the variation observed among specimens of *Curculionichthys*, those characters appear to be conserved enough to be used as synapomorphies and delimit this new genus of all remaining Otothyrinae.

### Description of three new species

#### Curculionichthys sabaji sp. n.

Taxon classificationAnimaliaSiluriformesLoricariidae

http://zoobank.org/48C22C5D-2C7E-4ED5-AD1C-C3DF6568F322

Figure 3 ; Table 1

##### Holotype.

MZUSP 117379, female, 23.3 mm SL, Pará State, municipality of Altamira, Rio 13 de Maio, tributary of Rio Curuá, Rio Iriri drainage, 08°43'41"S, 55°01'38"W, 22 October 2007, coll. Birindelli JLO, Netto-Ferreira AL, Sabaj-Perez MH, Lujan NK.

##### Paratypes.

All from Brazil, Rio Xingu basin. LBP 19763 (1, female, 23.4 mm SL), Pará State, municipality of Altamira, Rio Curuá, Rio Iriri drainage, 08°19'07"S, 55°05'23"W, 22 October 2007, coll. Birindelli JLO, Netto-Ferreira AL, Sabaj-Perez MH, Lujan NK. MZUSP 95711 (5, 16.3−20.0 mm SL, 2 c&s, sex not determined, 18.7−19.9 mm SL), Mato Grosso State, municipality of Gaúcha do Norte, Rio Coronel Vanick, 13°31'34"S, 52°43'52"W, 08 October 2007, coll. Lima FCT, Moreira CR, Ribeiro AC, Moraes L, Leite CMC. MZUSP 96959 (2, 19.1−20.7 mm SL), Pará State, municipality of Altamira, Rio 13 de Maio, tributary of Rio Curuá, Rio Iriri drainage, 08°38'53"S, 55°01'41"W, 22 October 2007, coll. Birindelli JLO, Netto-Ferreira AL, Sabaj-Perez MH, Lujan NK. MZUSP 97039 (5, 17.0−19.2 mm SL), Mato Grosso State, municipality of Campinápolis, Rio Couto de Magalhães, 13°48'02"S, 53°03'43"W, 10 October 2007, coll. Lima FCT, Moreira CR, Ribeiro AC, Moraes L, Leite CMC. MZUSP 97138 (1, 23.6 mm SL), collected with holotype. MZUSP 97198 (2, 20.0−22.3 mm SL), Pará State, municipality of Altamira, Rio Curuá, Rio Iriri drainage, 08°19'07"S, 55°05'23"W, 22 October 2007, coll. Birindelli JLO, Netto-Ferreira AL, Sabaj-Perez MH, Lujan NK.

**Figure 3.**
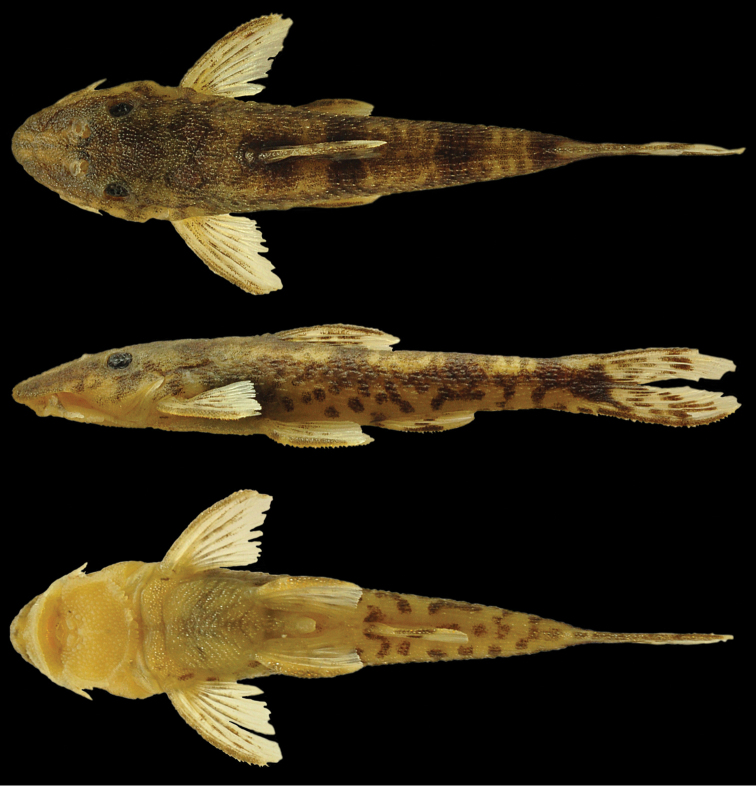
*Curculionichthys sabaji*, MZUSP 117379, holotype, female, 23.3 mm SL, from Pará State, municipality of Altamira, Rio 13 de Maio, Rio Xingu basin, 08°43'41"S 55°01'38"W.

##### Diagnosis.

**Figure 4.**
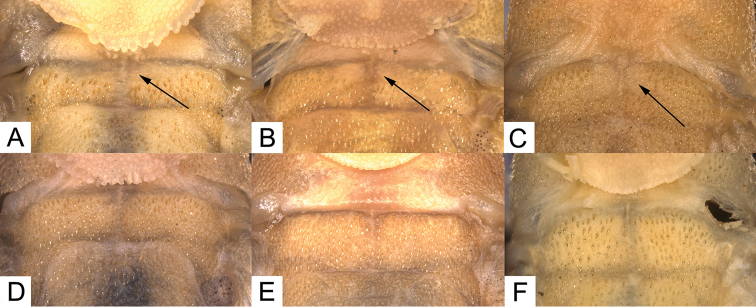
Photographs showing the cleithrum with an area free of odontodes (black arrow) in species of **A** *Curculionichthys sabaji*, MZUSP 117379, holotype, 23.3 mm SL **B** *Curculionichthys coxipone*, NUP 14947, paratype, 23.9 mm SL and **C** *Curculionichthys paresi*, LBP 13351, paratype, 23.4 mm SL; and the cleithrum completely covered with odontodes in **D** *Curculionichthys sagarana*, NUP 9714, paratype, 24.2 mm SL **E** *Curculionichthys oliveirai*, LBP 14917, paratype, 29.9 mm SL; and **F** *Curculionichthys insperatus*, LBP 6770, 25.0 mm SL.

**Figure 5.**
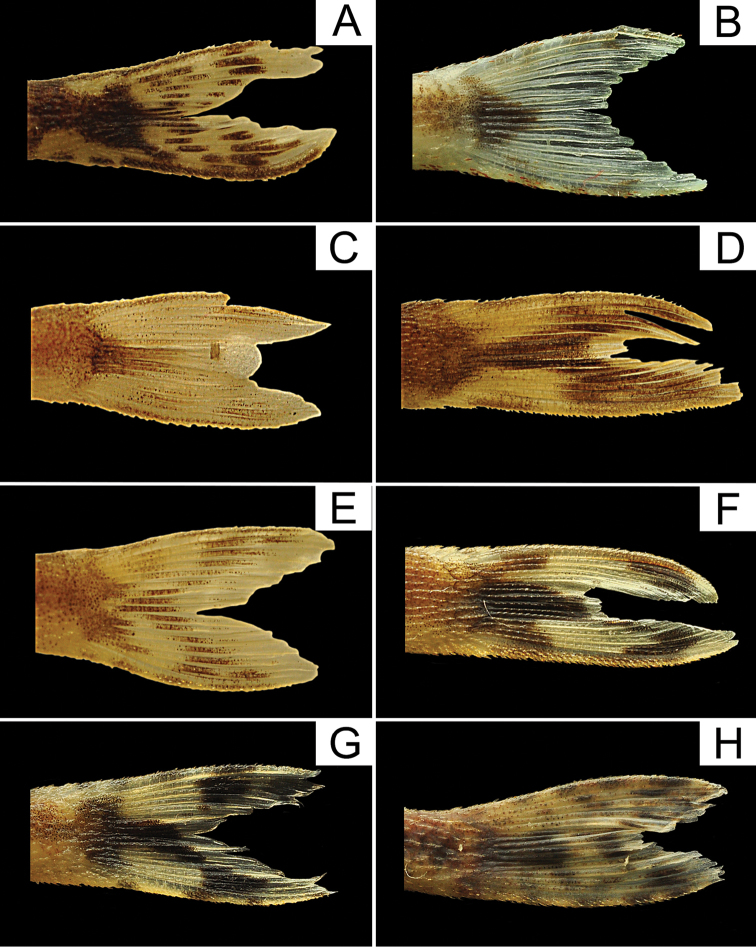
Coloration pattern of caudal fin of *Curculionichthys* species. **A** *Curculionichthys sabaji*, MZUSP 117379, holotype, 23.3 mm SL **B** *Curculionichthys insperatus*, LBP 17432, 26.9 mm SL **C** *Curculionichthys sagarana*, NUP 9715, paratype, 21.7 mm SL **D** *Curculionichthys coxipone*, MZUSP 117380, holotype, 29.0 mm SL **E** *Curculionichthys oliveirai*, LBP 13332, paratype, 23.8 mm SL **F** *Curculionichthys luteofrenatus*, LBP 19534, 30.5 mm SL **G** *Curculionichthys paresi*, LBP 13351, paratype, 24.6 mm SL **H** *Curculionichthys piracanjuba*, LBP 17256, 22.1 mm SL.

**Figure 6.**
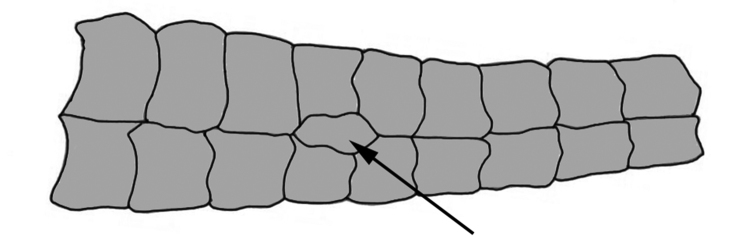
Diagram of dorsal view of the caudal peduncle of *Curculionichthys sagarana* showing the presence of one unpaired platelet on dorsal portion of caudal peduncle (black arrow).

*Curculionichthys sabaji* differs from all congeners by having several dark-brown spots distributed on the body (*vs.* a variety of pigment patterns, but none of which includes dark-brown spots). Moreover, the new species differs from all congeners, except *Curculionichthys coxipone* and *Curculionichthys paresi* by having the cleithrum with an area free of odontodes, Fig. 4A (*vs.* cleithrum completely covered with odontodes, Fig. 4D–F). The new species further differs from *Curculionichthys piracanjuba*, *Curculionichthys sagarana*, and *Curculionichthys oliveirai* by having some papillae of the lower lip arranged in a medial longitudinal series extending posterior to dentaries through the middle portion of the lower lip (*vs.* lower lip with all papillae randomly distributed); from *Curculionichthys coxipone* and *Curculionichthys oliveirai* by having the anterior profile of the head pointed (*vs.* rounded); from *Curculionichthys piracanjuba* by having odontodes forming longitudinally aligned rows on head and trunk (*vs.* odontodes not forming longitudinally aligned rows on head and trunk); from *Curculionichthys insperatus* and *Curculionichthys sagarana* by having the caudal fin hyaline, with one dark strip extending from caudal peduncle base to the median caudal fin rays, and for dark chromatophores irregular distributed almost forming two bands, Fig. 5A (*vs.* caudal fin hyaline, with dark blotch limited to caudal peduncle base, Fig. 5B and 5C respectively); from *Curculionichthys sagarana* by the absence of one unpaired platelet on the dorsal portion of caudal peduncle (*vs.* one unpaired platelet on the dorsal portion of the caudal peduncle, Fig. 6); from *Curculionichthys insperatus* by having small, inconspicuous odontodes forming rows on the head and trunk (*vs.* large, conspicuous odontodes forming rows on the head and the trunk); from *Curculionichthys oliveirai* by having 6−9 lateral abdomen plates (*vs.* 4−5 lateral abdomen plates); from *Curculionichthys paresi* by lacking contrasting dark geometric spots on the anterodorsal region of body (*vs.* presence of geometric spots); from *Curculionichthys piracanjuba* by not having hypertrophied odontodes on the snout tip (*vs.* hypertrophied odontodes on the snout tip). Additionally, *Curculionichthys sabaji* is distinguished by having a shorter dorsal fin spine (18.5−22.7% of SL, *vs.* 25.2−27.0% of SL in *Curculionichthys paresi*; 23.2−26.9% of SL in *Curculionichthys insperatus*); a shorter pectoral-fin spine (18.9−23.4% of SL, *vs.* 27.0−30.1% of SL in *Curculionichthys paresi*); a deeper caudal peduncle (7.0−10.0% of SL, *vs.* 10.8−12.5% of SL in *Curculionichthys oliveirai*; 10.2−11.3% of SL in *Hisonotus paresi*); a deeper head (40.9−49.1% of HL, *vs.* 51.6−59.2% of HL in *Curculionichthys oliveirai*); a longer head (34.3−38.6% of SL, *vs.* 27.9−32.2% of SL in *Curculionichthys piracanjuba*; 28.8−33.3% of SL in *Curculionichthys luteofrenatus*); a shorter snout (45.5−56.9% of HL, *vs.* 67.7−72.7% of HL in *Curculionichthys piracanjuba*; 67.0−75.3% of HL in *Curculionichthys luteofrenatus*) and a shorter interorbital width (30.3−35.7% of HL, *vs.* 36.7−40.9% of HL in *Curculionichthys piracanjuba*; 67.0−75.3% of HL in *Curculionichthys luteofrenatus*).

##### Description.

Morphometric and meristic data are given in Table 1. Small-size loricariid; maximum body length reached 23.6 mm SL. In lateral view, dorsal profile of body straight from snout tip to interorbital region; slightly convex to dorsal fin origin; and almost straight and decreasing to end of caudal peduncle. Ventral surface of body concave at tip of snout to anal fin insertion; concave to caudal fin insertion. Greatest body depth at dorsal fin origin. Greatest body width at opercular region; progressively narrowing towards snout and caudal fin. Trunk and caudal peduncle almost ellipsoid; rounded laterally and almost flat dorsally and ventrally.

**Table 1.** Morphometrics and meristic data for *Curculionichthys* species. SD = Standard deviation.

	*Curculionichthys sabaji*, n = 17					*Curculionichthys coxipone*, n = 38					*Curculionichthys sagarana*, n = 10				
	Holotype	Low	High	Mean	SD	Holotype	Low	High	Mean	SD	Holotype	Low	High	Mean	SD
SL (mm)	23.3	16.3	23.6	19.5	2.24	29.0	20.1	29.9	24.8	2.6	23.7	20.5	24.2	22.4	1.1
**Percents of SL**															
Head length	35.5	34.3	38.6	36.3	1.37	33.5	32.0	37.4	34.5	1.4	36.8	34.8	40.5	37.1	1.4
Predorsal length	47.4	41.1	47.7	44.5	1.87	44.2	42.6	51.6	45.6	1.9	46.9	40.1	49.3	46.5	2.6
Dorsal fin spine length	22.4	18.5	22.7	20.8	1.12	21.4	14.9	24.8	21.2	1.6	22.9	19.9	24.4	21.8	1.5
Anal fin unbranched ray length	17.9	13.5	20.1	16.6	1.86	22.5	18.0	22.5	20.4	1.0	18.8	16.6	20.5	18.5	1.2
Pectoral fin spine length	21.9	18.9	23.4	21.4	1.29	22.3	19.0	25.2	22.3	1.6	22.9	21.5	25.2	23.2	1.1
Pelvic fin unbranched ray length	18.6	15.1	19.2	17.3	1.13	20.9	17.4	25.4	21.3	1.9	19.1	16.2	23.5	19.9	2.3
Cleithral width	22.9	21.3	24.1	22.6	0.66	23.3	22.9	26.0	24.3	0.7	24.1	20.8	25.2	23.4	1.2
Thoracic length	17.4	12.3	22.7	15.1	2.90	16.5	14.6	23.9	16.6	1.4	19.2	14.8	19.4	17.2	1.5
Abdominal length	18.9	15.5	21.1	17.7	1.42	21.7	18.5	22.7	21.0	1.1	20.5	16.4	21.9	20.3	1.5
Caudal peduncle length	26.0	22.7	32.2	27.3	2.78	27.6	26.8	32.7	29.9	1.3	27.7	27.3	32.2	29.6	1.5
Caudal peduncle depth	7.9	7.0	10.0	8.7	0.83	10.1	8.8	10.9	10.1	0.4	9.6	8.4	9.6	9.2	0.4
**Percents of HL**															
Snout length	54.7	45.5	56.9	51.2	3.04	51.1	48.0	52.9	50.5	1.1	52.4	46.3	52.4	49.0	2.0
Orbital diameter	12.3	10.2	17.9	12.9	2.06	14.0	12.0	16.4	13.9	1.0	15.1	13.8	16.3	15.0	0.6
Interorbital width	32.7	30.3	35.7	32.0	1.24	35.6	33.8	37.8	36.0	1.1	31.9	27.4	33.6	31.3	2.0
Head depth	41.4	40.9	49.1	43.5	2.39	51.1	43.4	53.5	48.6	2.3	48.5	41.2	49.1	45.9	2.4
Suborbital depth	20.5	15.1	21.2	18.4	1.78	22.8	19.4	27.3	22.7	1.6	20.7	16.9	21.1	19.5	1.3
Mandibular ramus	8.6	2.9	8.66	5.0	1.55	10.8	8.2	12.5	10.0	1.0	9.7	6.6	9.7	8.7	0.9
	**Holotype**	**Low**	**High**	**Mode**	**SD**	**Holotype**	**Low**	**High**	**Mode**	**SD**	**Holotype**	**Low**	**High**	**Mode**	**SD**
**Meristics**															
Left lateral scutes	24	24	25	24	-	14	25	27	26	-	16	24	25	24	-
Left premaxillary teeth	12	7	12	7	-	11	7	15	13	-	16	15	19	16	-
Left dentary teeth	9	5	12	7	-	8	7	16	12	-	14	12	18	13	-

**Table 2.** Species reallocated from *Hisonotus* to the newly described genus *Curculionichthys*.

Original description	New generic allocation
*Hisonotus insperatus* Britski & Garavello, 2003	*Curculionichthys insperatus* (Britski & Garavello, 2003)
*Hisonotus luteofrenatus* Britski & Garavello, 2007	*Curculionichthys luteofrenatus* (Britski & Garavello, 2007)
*Hisonotus oliveirai* Roxo, Zawadzki & Troy, 2014	*Curculionichthys oliveirai* (Roxo, Zawadzki & Troy, 2014)
*Hisonotus paresi* Roxo, Zawadzki & Troy, 2014	*Curculionichthys paresi* (Roxo, Zawadzki & Troy, 2014)
*Hisonotus piracanjuba* Martins & Langeani, 2012	*Curculionichthys piracanjuba* (Martins & Langeani, 2012)

Head elliptical in dorsal view; snout long (45.5−56.9% HL), slightly pointed, its tip rounded, flat to slightly convex between orbits. Dorsal and ventral series of odontodes completely covering anterior margin of snout; odontodes of snout slightly larger in size than remaining ones found on head. Snout tip completely covered with odontodes. Odontodes on head and trunk well defined and arranged into longitudinal rows (one odontode after the other, but not necessarily forming parallel series). Eye small and round (10.2−17.9% HL), situated dorsolaterally in midpoint of head. Iris operculum present but poorly developed. No ridge between eyes and nares. Nostril small. Supraoccipital process not elevated and without tuft of odontodes in specimens of all size. Mouth wide; oral disk roundish with papillae arranged in a medial longitudinal series extending posterior to dentaries through middle portion of lower lip. Lower lip larger than upper; almost reaching cleithrum region; its border strongly fringed. Maxillary barbel short, slender and free distally. Teeth slender and bicuspidate. Cusps symmetrical; medial cusp larger than lateral. Premaxillary teeth 7–12. Dentary teeth 5–12.

**Figure 7.**
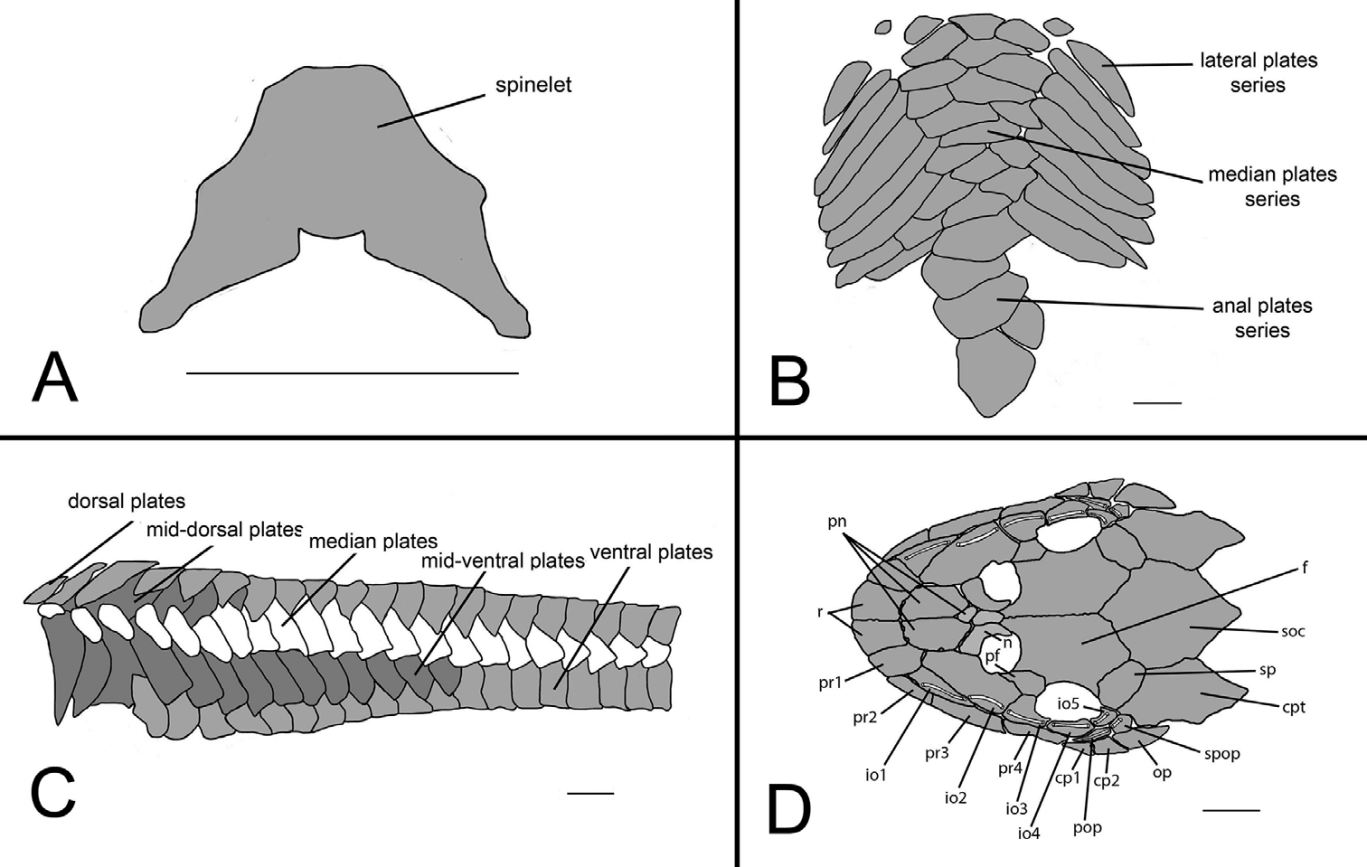
*Curculionichthys sabaji*, MZUSP 95711, 19.9 mm SL **A** Spinelet shape; **B** Ventral view of abdominal plates **C** Lateral trunk plates **D** Cranial bones plates of the head in dorsal view. Scale bar: 0.5 mm (**A**); 1 mm (**B, C, D**).

Dorsal fin rays ii, 7; in lateral view dorsal fin originating slightly posterior through origin of pelvic fin; distal margin slightly convex. Tip of adpressed dorsal fin rays surpassing end of anal fin base. Dorsal fin spinelet short and V-shaped (Fig. 7A); lock mechanism functional. Pectoral fin rays i, 6; tip of longest tip of longest pectoral-fin ray almost reaching pelvic fin insertion, when adpressed. Pectoral axillary slit present between pectoral fin insertion and lateral process of cleithrum. Pelvic fin rays i, 5; distal margin slightly convex; tip of adpressed pelvic fin almost reaching anal fin origin. Adipose-fin absent. Anal fin rays i, 4; distal margin slightly convex. Caudal fin rays i, 7-7, i; slightly emarginate; both unbranched rays of same size. Adpressed rays of all fins covered with pointed odontodes. Total vertebrae 28.

Body completely covered by bony plates, except on ventral part of head, around pectoral and pelvic fin origins and on dorsal fin base. Abdomen entirely covered by plates (Fig. 7B), abdomen formed by lateral plate series with elongate and large plates, formed by two lateral plates series, similar in size; median plates formed by one to three plates series reaching anal shield. Lateral of body entirely covered by plates (Fig. 7C); mid-dorsal plates poorly developed, almost reaching end of dorsal fin base; median plates not interrupted in median portion of body; mid-ventral plates almost reaching middle of caudal peduncle. Cleithrum and coracoid totally exposed. Arrector fossae partially enclosed by ventral lamina of coracoids.

Parts of dorsal head bone plates presented in Fig. 7D. Snout tip formed by one pair of rostral square-shaped plates (r). Nasal (n) almost rectangular forming anterior medial nostril margin in contact posteriorly with frontals (f) and anteriorly and laterally with pre-nasals (pn). Pre-nasals (pn) positioned posteriorly of rostral plates (r), formed by two large and two small rounded-shaped plates between nares. Top of head composed by compound pterotic (cpt), parieto supraoccipital (soc) and frontal (f), largest bones of head, and prefrontal (pf) and sphenotic (sp). Compound pterotic (cpt) fenestrated randomly distributed. Posterior rostrum plates pr1-pr2 small, and rectangular shaped; pr4-pr3 largest, and rectangular shaped. Infraorbital plate series complete (io1-io5), present just above posterior rostrum series, all covered by latero-sensory canal system; io2 largest and io5 smallest; io3, io4 and io5 forming inferior orbital margin of eyes; preopercle (pop) elongated and rectangular, covered by latero-sensory canal; preopercle present under io4 and io5, and upper cp1, cp2. Supra-opercular plate (spop) present just above preopercle, covered by latero-sensory canal. Subocular cheek plates (cp1-cp2) and opercle (op) form posterior lateral margin of head.

**Color in alcohol.** Ground color of dorsal and ventral region of head and trunk pale yellowish; dorsal portion darker than ventral. Four dark saddle along dorsal portion of body: one at dorsal fin origin; second at end of dorsal fin; third at middle of caudal peduncle; and fourth at upper caudal peduncle adpressed ray origin. Dorsal end ventral surface covered with small dark-dots smaller then eyes diameter. Unpigmented portion of snout appears as two hyaline parallel stripes from rostral plate to nares. Dorsal, pectoral, and pelvic fins with dark chromatophores forming irregular sets of bands: three on dorsal and pectoral fin, two on pelvic fin and one on anal fin. Caudal fin hyaline, except for dark stripe on origin of rays, and for dark chromatophores irregularly distributed forming two diffuse bands.

**Sexual dimorphism.** Adults males have a papilla in urogenital opening (*vs.* absent in females); have a long pelvic fin that extends beyond anal fin origin (*vs.* pelvic fin not reaching anal fin origin in females); and have an unbranched pelvic fin ray supporting a dermal flap along its dorsal surface. Both sexes have a membrane on anal opening; however, this membrane is more developed in females than in males, covering almost the entire urogenital opening (see reference to this last character in Roxo et al. 2014b).

##### Distribution.

**Figure 8.**
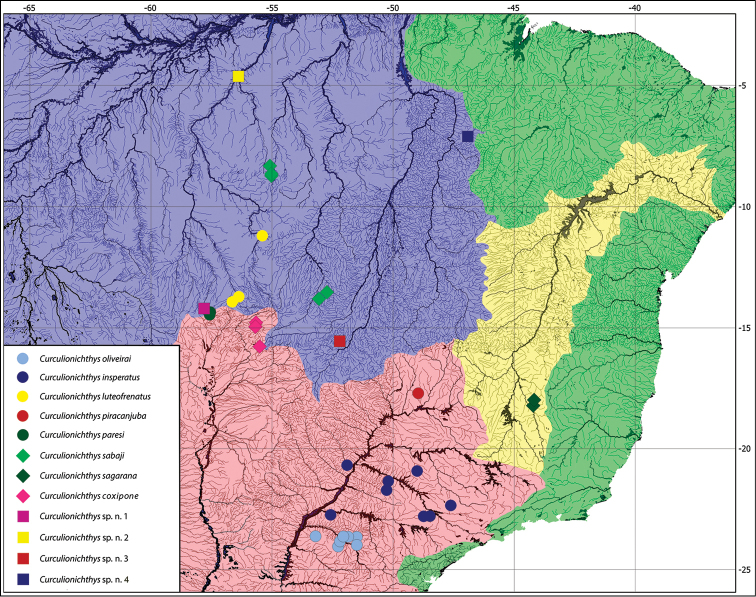
Map showing the distribution of *Curculionichthys* species. Red – La Plata basin; Blue – Amazon basin; Yellow – Rio São Francisco basin; Green – Coastal Drainages to Atlantic.

The new species *Curculionichthys sabaji* are known from five localities in the Rio Xingu basin: two at Rio 13 de Maio, one at Rio Coronel Vanick, one at Rio Couto de Magalhães and one at Rio Curuá (Fig. 8).

##### Etymology.

The specific name “sabaji” is a patronym honoring Dr. Mark Henry Sabaj Pérez, Collection Manager of Ichthyology, Academy of Natural Sciences of Philadelphia, in recognition of his dedication and contributions to study of Neotropical fishes especially from Rio Xingu basin (iXingu Project).

##### Comparative remarks.

*Curculionichthys sabaji* from the Xingu basin is morphologically very similar to *Curculionichthys paresi* from Rio Paraguai basin. Both species share a low number of teeth in the premaxillaries and dentaries, the form of papillae in the lower lip and the general pattern of body coloration. However, *Curculionichthys sabaji*, can be distinguished from *Curculionichthys paresi* by having several dark-brown spots distributed on the body, a shorter dorsal fin spine, a shorter pectoral fin spine, a deeper caudal peduncle and the lack of dark geometric spots on the anterodorsal region of body. The similarity in morphology among both species suggests a close relationship between them and that they may have once shared a common ancestor. Furthermore, the presence of these close related species in the Rio Paraguay and the Rio Xingu is not a surprise, since several authors (e.g. Pearson 1937; Carvalho and Albert 2011) historically have reported that those two hydrographic systems share several lineages of fishes, and that most species lineage present in the Rio Paraguay originated in Amazonian drainages (Carvalho and Albert 2011).

#### Curculionichthys coxipone sp. n.

Taxon classificationAnimaliaSiluriformesLoricariidae

http://zoobank.org/66B213A7-69B9-4980-B4EB-B7AFEEE43D5F

Figure 9 ; Table 1

Hisonotus sp. 5 – Roxo et al. 2014a: 9(8) e105564 (phylogenetic relationships).

##### Holotype.

MZUSP 117380, female, 29.0 mm SL, Mato Grosso State, municipality of Cuiabá, tributary of Rio Aricá Mirim, Rio Cuiabá drainage, Rio Paraguai basin, 15°46'03"S, 55°30'44"W, September 2011, coll. Mehanna MN, Ferreira AT.

##### Paratypes.

All from Brazil, Mato Grosso State, Rio Cuiabá drainage, Rio Paraguai basin. LBP 5061 (3 females, 21.7−30.0 mm SL, 2 males, 25.8−27.9 mm SL), municipality of Cuiabá, tributary of Rio Aricá Mirim, 15°46'03"S, 55°30'44"W, 07 September 2007, coll. Mehanna MN, Ferreira AT. LBP 5062 (3 females, 22.5−28.7 mm SL), municipality of Cuiabá, tributary of Rio Aricá Mirim, 15°46'03"S, 55°30'44"W, 07 September 2007, coll. Mehanna MN, Ferreira AT. LBP 5069 (9 females, 22.5−29.6 mm SL, 3 males, 25.6−26.9 mm SL, 1 c&s, sex not determined, 25.6 mm SL), municipality of Cuiabá, tributary of Rio Aricá Mirim, 15°46'03"S, 55°30'44"W, 08 November 2007, coll. Mehanna MN, Ferreira AT. LBP 5646 (11 females, 21.8−28.8 mm SL, 7, males, 24.9−28.0 mm SL, 3 c&s, sex not determined, 26.8−28.2 mm SL), municipality of Cuiabá, tributary of Rio Aricá Mirim, 15°46'03"S, 55°30'44"W, 11 November 2007, coll. Mehanna MN, Ferreira AT. NUP 2264 (6 females, 18.2−25.3 mm SL, 6 males, 23.4−23.7 mm SL), municipality of Chapada dos Guimarães, Córrego São Joaquim, 14°46'53"S, 55°39'57"W, 26 March 2014, coll. NUPELIA´s team. NUP 14947 (6 females, 21.2−25.1 mm SL, 21.9−25.0 mm SL, 3 juveniles), municipality of Chapada dos Guimarães, Córrego Laranjinha, tributary of Rio Manso, 14°57'18"S, 55°41'15"W, June 2013, coll. NUPELIA´s team. NUP 16442 (6 females, 23.4−28.7 mm SL, 1 c&s sex not determined, 28.7 mm SL), collected with holotype.

**Figure 9.**
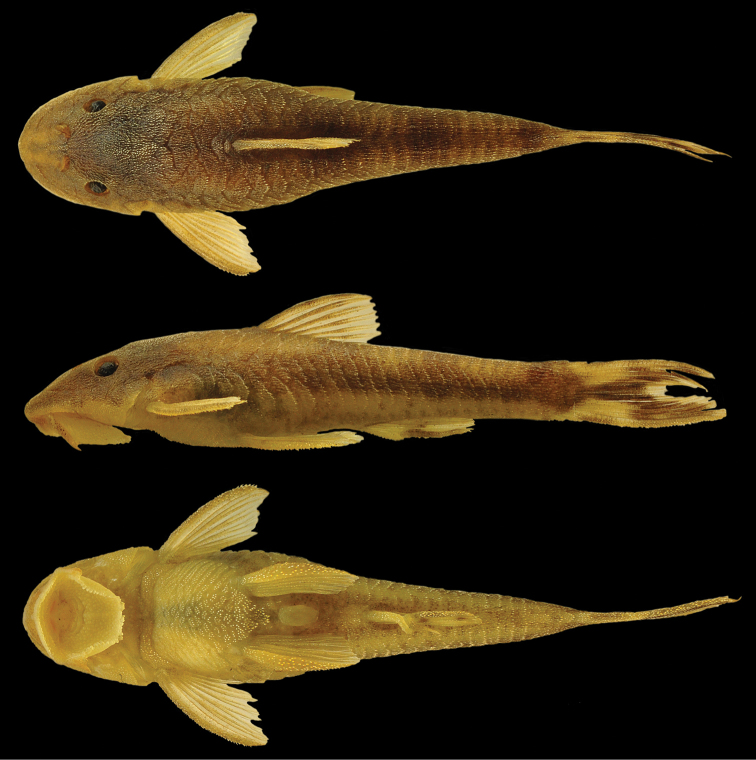
*Curculionichthys coxipone*, MZUSP 117380, holotype, female, 29.0 mm SL, from Mato Grosso State, municipality of Cuiabá, tributary of Rio Aricá Mirim, Rio Cuiabá drainage, 15°46'03"S, 55°30'44"W.

##### Diagnosis.

*Curculionichthys coxipone* differs from all congeners by having a higher number of vertebrae 29−30 (*vs.* 28 in all other species of *Curculionichthys*). The new species differs from all congeners, except *Curculionichthys sabaji* and *Curculionichthys paresi* by having the cleithrum with an area free of odontodes, Fig. 4B (*vs.* cleithrum completely covered with odontodes, Fig. 4D−F). The new species further differs from all congeners, except *Curculionichthys oliveirai* by having the anterior profile of the head rounded (*vs.* pointed); from *Curculionichthys piracanjuba*, *Curculionichthys sagarana*, and *Curculionichthys oliveirai* by having lower lip with some papillae arranged in a medial longitudinal series extending posterior to dentaries through middle portion of lower lip (*vs.* lower lip with all papillae randomly distributed); from *Curculionichthys insperatus* and *Curculionichthys oliveirai* by having the caudal fin hyaline, with one dark stripe extending from the caudal peduncle base to the middle caudal fin rays, and dark chromatophores irregular distributed almost forming one band, Fig. 5D (*vs.* caudal fin hyaline, with dark blotch limited to caudal peduncle base, Fig. 5B and E, respectively); from *Curculionichthys paresi* by lacking contrasting dark-brown geometric spots on the anterior region of the body (*vs.* presence of dark-brown geometric spots); from *Curculionichthys sabaji* by lacking several dark-brown spots distributed on the body (*vs.* presence of dark-brown spots); from *Curculionichthys oliveirai* and *Curculionichthys coxipone* by having the anterior profile of the head pointed (*vs.* rounded); from *Curculionichthys oliveirai* by having 7−9 lateral abdomen plates (*vs.* 4−5 lateral abdomen plates); from *Curculionichthys paresi* by having more dentary teeth 9−13 (*vs.* 4−7); from *Curculionichthys oliveirai* by having 6−9 lateral abdomen plates (*vs.* 4−5 lateral abdomen plates); from *Curculionichthys sagarana* by absence of one unpaired platelets on dorsal portion of caudal peduncle (*vs.* presence of one unpaired platelets on dorsal portion of caudal peduncle, Fig. 6); from *Curculionichthys piracanjuba* by having some papillae on the lower lip arranged in a medial longitudinal series extending posterior to the dentaries through the middle portion of lower lip (*vs.* lower lip with all papillae randomly distributed) and by not having hypertrophied odontodes on the snout tip (*vs.* hypertrophied odontodes on the snout tip); from *Curculionichthys insperatus* by having small, inconspicuous odontodes forming rows on the head and trunk (*vs.* large, conspicuous odontodes forming rows on the head and the trunk). Additionally, *Curculionichthys coxipone* is distinguished by having a shorter interorbital distance (33.8−37.8% of HL, *vs.* 27.4−33.6% of HL in *Curculionichthys sagarana*); a shorter dorsal fin spine (14.9−24.8% of SL, *vs.* 25.2−27.0% of SL in *Curculionichthys paresi*); a shorter pectoral fin spine (19.0−25.2% of SL, *vs.* 27.0−30.1% of SL in *Curculionichthys paresi*); a longer mandibular ramus (8.2−12.5% of HL, *vs.* 6.0−8.0% of HL in *Curculionichthys paresi*); and a shorter snout (48.0−58.9% of HL, *vs.* 67.7−72.7% of HL in *Curculionichthys piracanjuba*; 67.0−75.3% of HL in *Curculionichthys luteofrenatus*).

##### Description.

Morphometric and meristic available in Table 1. Small loricariid; bigger specimen examined reached 29.9 mm SL. In lateral view, dorsal profile of head convex from snout tip to posterior margin of parieto supraoccipital, and straight to dorsal fin origin. Dorsal profile of trunk slightly concave and descending from dorsal fin origin to end of dorsal fin base, straight to caudal peduncle. Ventral profile concave from snout tip to opercular region; convex from opercular region to anal fin origin; concave to caudal fin insertion. Greatest body depth at dorsal fin origin. Greatest body width at opercular region, gradually decreasing towards snout and caudal fin. Cross-section of trunk and caudal peduncle almost ellipsoid; rounded laterally and almost flat dorsally and ventrally.

Head rounded in dorsal view; snout round to slightly pointed, its tip rounded, elongated (48.0−52.9% HL), slightly convex between orbits. Dorsal and ventral series of odontodes along anterior margin of snout completely covering its tip; odontodes at same size than remaining ones on head. Odontodes on head and trunk hypertrophied and arranged in longitudinal rows (most prominent on head). Eyes moderately small (12.0−16.4% HL), dorsolaterally positioned. Lips roundish with papillae arranged in a medial longitudinal series extending posterior to dentaries through middle portion of lower lip. Lower lip larger than upper lip; its border fringed. Maxillary barbel present; joined to lower lip. Teeth slender and bicuspid; medial cusp larger than lateral cusp. Premaxillary teeth 7−15. Dentary teeth 7−16.

**Figure 10.**
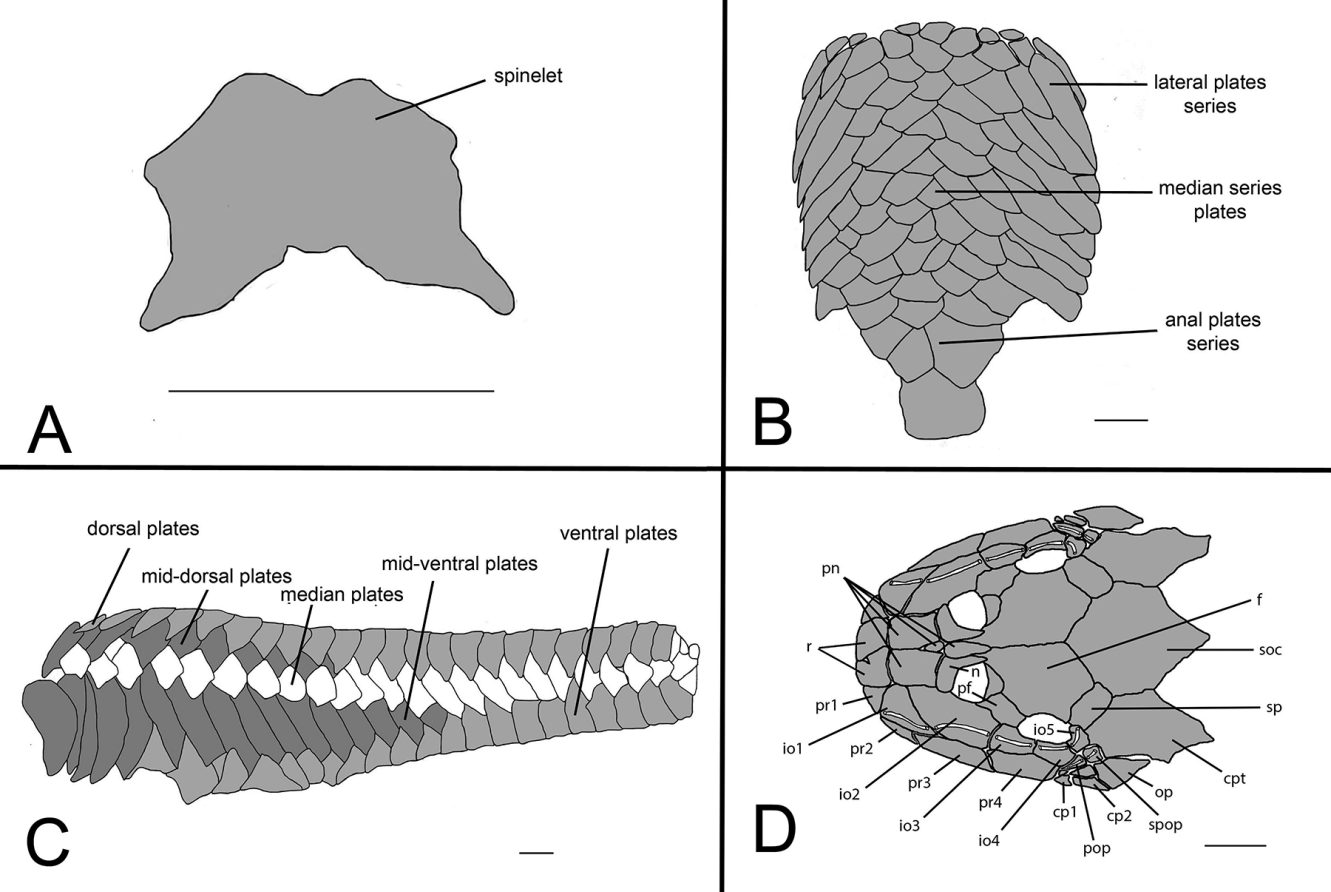
*Curculionichthys coxipone*, LBP 5646, 27.5 mm SL. **A** Spinelet shape **B** Ventral view of abdominal plates **C** Lateral trunk plates **D** Cranial bones plates of the head in dorsal view. Scale bar: 0.5 mm (**A**); 1 mm (**B, C, D**).

Dorsal fin ii, 7; dorsal fin spinelet short and V-shaped (Fig. 10A); dorsal fin lock functional; dorsal fin origin slightly posterior to pelvic fin origin. Tip of adpressed dorsal fin reaching anal fin insertion. Pectoral fin i, 6; its tip reaching beyond pelvic fin insertion when depressed. Presence of pectoral axillary slit between pectoral fin insertion and lateral process of cleithrum variable; absent in some specimens. Pectoral spine supporting odontodes on ventral, anterior and dorsal surfaces. Pelvic fin i, 5; tip of pelvic fin unbranched ray almost reaching anal fin origin when depressed in females and reaching anal fin origin in males. Pelvic fin unbranched ray with dermal flap along dorsal surface in males. Anal fin i, 5; distal margin slightly convex. Caudal fin i, 7-7, i; slightly emarginate; both unbranched rays of same size. Adipose fin absent. Total vertebrae 29−30 (1 c&s 29 vertebrae and 3 c&s 30 vertebrae).

Body covered with bony plates, except above head, around pectoral and pelvic fin origins and on dorsal fin base. Cleithrum and coracoid partially exposed. Arrector fossae partially to completely enclosed by ventral lamina of coracoids. Abdomen entirely covered by plates (Fig. 10B); lateral plates series with elongated and large plates formed by two lateral plate series, similar in size; median plates formed by six to seven irregular plate series reaching anal shield and lateral plate series; anal plates series covered by large square plates. Body entirely covered laterally by plates (Fig. 10C); mid-dorsal plates poorly developed and reaching middle of dorsal fin base; median plates series continuous in median portion of body; mid-ventral plates reaching of caudal peduncle origin.

Parts of dorsal head bone plates presented in Fig. 10D. Snout tip formed by one pair of rostral rectangular-shaped plates (r). Nasal (n) almost rectangular forming anterior medial nostril margin in contact posteriorly with frontals (f) and anteriorly and laterally with pre-nasals (pn). Pre-nasals (pn) positioned posteriorly of rostral plates (r), formed by two large and one small oval-shaped plates, and one elongate oval shaped between nares. Top of head composed by compound pterotic (cpt), parieto supraoccipital (soc) and frontal (f), largest bones of head, and prefrontal (pf) and sphenotic (sp). Compound pterotic (cpt) fenestrated randomly distributed. Posterior rostrum plates pr1-pr2 small, first triangular and second rectangular-shaped; pr4-pr3 largest, and rectangular shaped. Infraorbital plate series complete (io1-io5), present just above posterior rostrum series, all covered by latero-sensory canal system; io2 largest and io5 smallest; io3, io4 and io5 forming inferior orbital margin of eyes; preopercle (pop) elongated and rectangular, covered by latero-sensory canal; preopercle present under io4, and upper cp1, cp2 and op. Supra-opercular plate (spop) present just above preopercle, covered by latero-sensory canal. Subocular cheek plates (cp1-cp2) and opercle (op) form posterior lateral margin of head.

**Color in alcohol.** Ground color of dorsal and ventral region of head and trunk pale yellowish; dorsal portion darker than ventral. Four dark saddle along dorsal portion of body: first at dorsal fin origin; second at end of dorsal fin; third at middle of caudal peduncle; and fourth at end of caudal peduncle. Unpigmented portion of snout appears as two hyaline parallel stripes from rostral plate to nares. Dorsal, pectoral, and pelvic fins hyaline. Caudal fin hyaline, with dark stripe extending from caudal peduncle base onto base of median caudal fin rays, and with dark chromatophores forming one large band.

**Sexual dimorphism.** Adults males have a papilla in urogenital opening (*vs.* absent in females); and have an unbranched pelvic fin ray supporting a dermal flap along its dorsal surface. Both sexes have a membrane on the anal opening; however, this membrane is more developed in females than in males, covering almost the entire urogenital opening (see reference to this last character in Roxo et al. 2014b).

##### Distribution.

The new species *Curculionichthys coxipone* is known from Rio Cuiaba drainage, Rio Paraguay basin, Mato Grosso State in Brazil (Fig. 8).

##### Etymology.

The specific name “coxipone” refers to the Coxiponé indigenous people who inhabit the margins of Rio Cuiabá, near to the municipality of Cuiabá in Mato Grosso State, Brazil. A noun in opposition.

##### Comparative remarks.

*Curculionichthys coxipone* is similar in external morphology with *Curculionichthys oliveirai* from Rio Ivaí, upper Rio Paraná basin. However, the new species *Curculionichthys coxipone* can be distinguished from *Curculionichthys oliveirai* by having the cleithrum with an area free of odontodes, a higher number of vertebrae 29−30 and by a hyaline caudal fin, with one dark stripe extending from the caudal peduncle base to the median caudal fin rays, and for dark chromatophores irregular distributed almost forming one band. Furthermore, the presence of a higher number of vertebrae appears to be unique to *Curculionichthys coxipone*.

#### Curculionichthys sagarana sp. n.

Taxon classificationAnimaliaSiluriformesLoricariidae

http://zoobank.org/DA95A052-B969-4650-BE03-683303C644D0

Figure 11 ; Table 1

##### Holotype.

MZUSP 117381, female 23.7 mm SL, Minas Gerais State, municipality of Santo Hipólito, Rio Pardo Grande, Rio das Velhas drainage, Rio São Francisco basin, 18°13'43"S, 44°13'03"W, 17 September 2007, coll. Leal CG, Junqueira NT, Pompeu PS.

##### Paratypes.

All from Brazil, Minas Gerais State, Rio das Velhas drainage, Rio São Francisco basin: LBP 19983 (1 male, 21.9 mm SL), municipality of Santo Hipólito, Rio Pardo Grande, 18°13'43"S, 44°13'03"W, 11 September 2007, coll. Leal CG, Junqueira NT, Pompeu PS. NUP 9714 (1 female, 24.4 mm SL, 1 male, 22.5 mm SL), municipality of Augusto de Lima, Rio Curimataí, 17°59'33"S, 44°10'48"W, 23 March 2008, coll. Leal CG, Junqueira NT, Pompeu PS. NUP 9715 (2 females, 17.5−18.4 mm SL, 1 male, 21.7 mm SL, 1 c&s sex not determined, 23.3 mm SL), municipality of Santo Hipólito, Rio Pardo Grande, 18°13'43"S, 44°13'03"W, 25 March 2010, coll. Leal CG, Junqueira NT, Pompeu PS. NUP 9716 (4 juveniles, sex not determined, 10.5−17.1 mm SL), municipality of Santo Hipólito, Rio Pardo Grande, 18°13'43"S, 44°13'03"W, 25 March 2010, coll. Leal CG, Junqueira NT, Pompeu PS. NUP 12595 (1 male, 23.0 mm SL), collected with holotype. NUP 12596 (1 female, 24.1 mm SL), municipality of Santo Hipólito, Rio Pardo Grande, 18°13'43"S, 44°13'03"W, 24 March 2008, coll. Leal CG, Junqueira NT, Pompeu PS. NUP 12597 (1 male, 21.7 mm SL), municipality of Santo Hipólito, Rio Pardo Grande, 18°13'43"S, 44°13'03"W, 24 March 2008, coll. Leal CG, Junqueira NT, Pompeu PS. NUP 12614 (1 female, 21.7 mm SL), municipality of Santo Hipólito, Rio Pardo Grande, 18°13'43"S, 44°13'03"W, 11 September 2007, coll. Leal CG, Junqueira NT, Pompeu PS.

**Figure 11.**
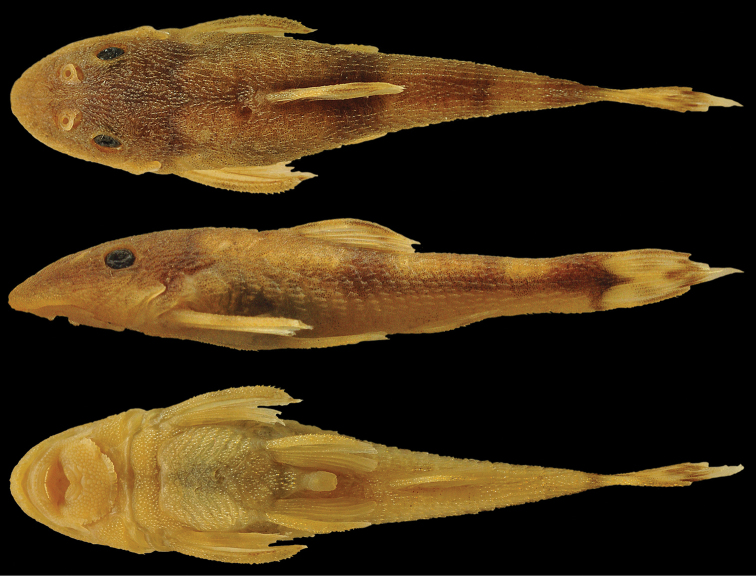
*Curculionichthys sagarana*, MZUSP 117381, holotype, female, 23.7 mm SL, from Minas Gerais State, municipality of Santo Hipólito, Rio Pardo Grande, tributary of Rio das Velhas, Rio São Francisco basin, 18°13'43"S, 44°13'03"W.

##### Diagnosis.

*Curculionichthys sagarana* differs from all congeners by having one unpaired platelet on the dorsal portion of the caudal peduncle, Fig. 6 (*vs.* dorsal portion of caudal peduncle without unpaired platelets). The new species can be further distinguished from all congeners, except *Curculionichthys insperatus* and *Curculionichthys luteofrenatus* by having the caudal fin hyaline, with dark blotch limited to caudal peduncle base, Fig. 5C (*vs.* caudal fin hyaline, with one dark stripe extending from caudal peduncle base to the middle caudal fin rays, and for dark chromatophores irregularly distributed almost forming one or two bands); from *Curculionichthys insperatus*, *Curculionichthys paresi* and *Curculionichthys sabaji* by having more premaxillary teeth 15−19 (*vs.* 10−12 in *Curculionichthys insperatus*; 6−10 in *Curculionichthys paresi* and 7−12 in *Curculionichthys sabaji*) and more dentary teeth 12−18 (*vs.* 8−12 in *Curculionichthys insperatus*, 4−7 in *Curculionichthys paresi* and 7−12 in *Curculionichthys sabaji*); from all congeners, except *Curculionichthys piracanjuba* and *Curculionichthys oliveirai*, by having all papillae on the lower lip randomly distributed (*vs.* lower lip with some papillae arranged in a medial longitudinal series extending posterior to dentaries through middle portion of lower lip); from *Curculionichthys oliveirai* and *Curculionichthys coxipone* by having the anterior profile of the head pointed (*vs.* rounded); from *Curculionichthys paresi* by lacking contrasting dark-brown geometric spots on the anterodorsal region of the body (*vs.* presence); from *Curculionichthys piracanjuba* by having odontodes forming longitudinally aligned rows on the head and trunk (*vs.* odontodes not forming longitudinally aligned rows on the head and trunk); from *Curculionichthys sabaji*, *Curculionichthys coxipone* and *Curculionichthys paresi* by having the cleithrum completely covered with odontodes, Fig. 4D (*vs.* the cleithrum with an area free of odontodes, Fig. 4A−C); from *Curculionichthys insperatus* by having small, inconspicuous odontodes forming rows on the head and trunk (*vs.* large, conspicuous odontodes forming rows on the head and the trunk); from *Curculionichthys oliveirai* by having 6−9 lateral abdomen plates (*vs.* 4−5 lateral abdomen plates); from *Curculionichthys piracanjuba* by not having hypertrophied odontodes on the snout tip (*vs.* hypertrophied odontodes on the snout tip). Additionally, *Curculionichthys sagarana* is distinguished by having a deeper caudal peduncle (8.4−9.6 % of SL, *vs.* 10.8−12.5% of SL in *Curculionichthys oliveirai*; 10.2−11.3% in *Curculionichthys paresi*); a greater head length (34.8−40.5% of SL, *vs.* 28.8−33.3% of SL in *Curculionichthys luteofrenatus*; 27.9−32.2% of SL in *Curculionichthys piracanjuba*); a shorter snout (46.3−52.4% of HL, *vs.* 67.0−75.3% of HL in *Curculionichthys luteofrenatus*; 67.7−72.7% of HL in *Curculionichthys piracanjuba*); a shorter interorbital width (27.4−33.6% of SL, *vs.* 33.3−45.4% of HL in *Curculionichthys luteofrenatus*; 36.7−40.9% of HL in *Curculionichthys piracanjuba*; 33.8−37.8% of HL in *Curculionichthys coxipone*); a deeper head (41.2−49.1% of HL, *vs.* 51.6−59.2% of HL in *Curculionichthys oliveirai*); a shorter dorsal-spine (19.9−24.4% of SL, *vs.* 25.2−27.0% of SL in *Curculionichthys paresi*); and a shorter pectoral-spine (21.5−25.2% of SL, *vs.* 27.0−30.1% of SL in *Curculionichthys paresi*).

##### Description.

Morphometric and meristic available in Table 1. Small loricariid; largest examined specimen reaching 24.2 mm SL. In lateral view, dorsal profile of head convex from snout tip to posterior margin of parietosupraoccipital, and straight to dorsal fin origin. Dorsal profile of trunk slightly concave and descending from dorsal fin origin to end of dorsal fin base, straight to caudal peduncle. Ventral profile concave from snout tip to opercular region; convex from opercular region to anal fin origin; concave to caudal fin insertion. Greatest body depth at dorsal fin origin. Greatest body width at opercular region, gradually decreasing towards snout and caudal fin. Cross-section of trunk and caudal peduncle almost ellipsoid; rounded laterally and almost flat dorsally and ventrally.

Head elliptical in dorsal view; snout round to slightly pointed, its tip rounded, elongated (46.3−52.4% HL), slightly convex between orbits. Dorsal and ventral series of odontodes along anterior margin of snout completely covering its tip; odontodes at same size than remaining ones on head. Odontodes on head and trunk hypertrophied and arranged in longitudinal rows (most prominent on head). Eyes moderately small (13.8−16.3% HL), dorsolaterally positioned. Lips roundish with papillae arranged in a medial longitudinal series extending posterior to dentaries through middle portion of lower lip. Lower lip larger than upper lip; its border fringed. Maxillary barbel present; joined to lower lip. Teeth slender and bicuspid; medial cusp larger than lateral cusp. Premaxillary teeth 15−19. Dentary teeth 12−18.

**Figure 12.**
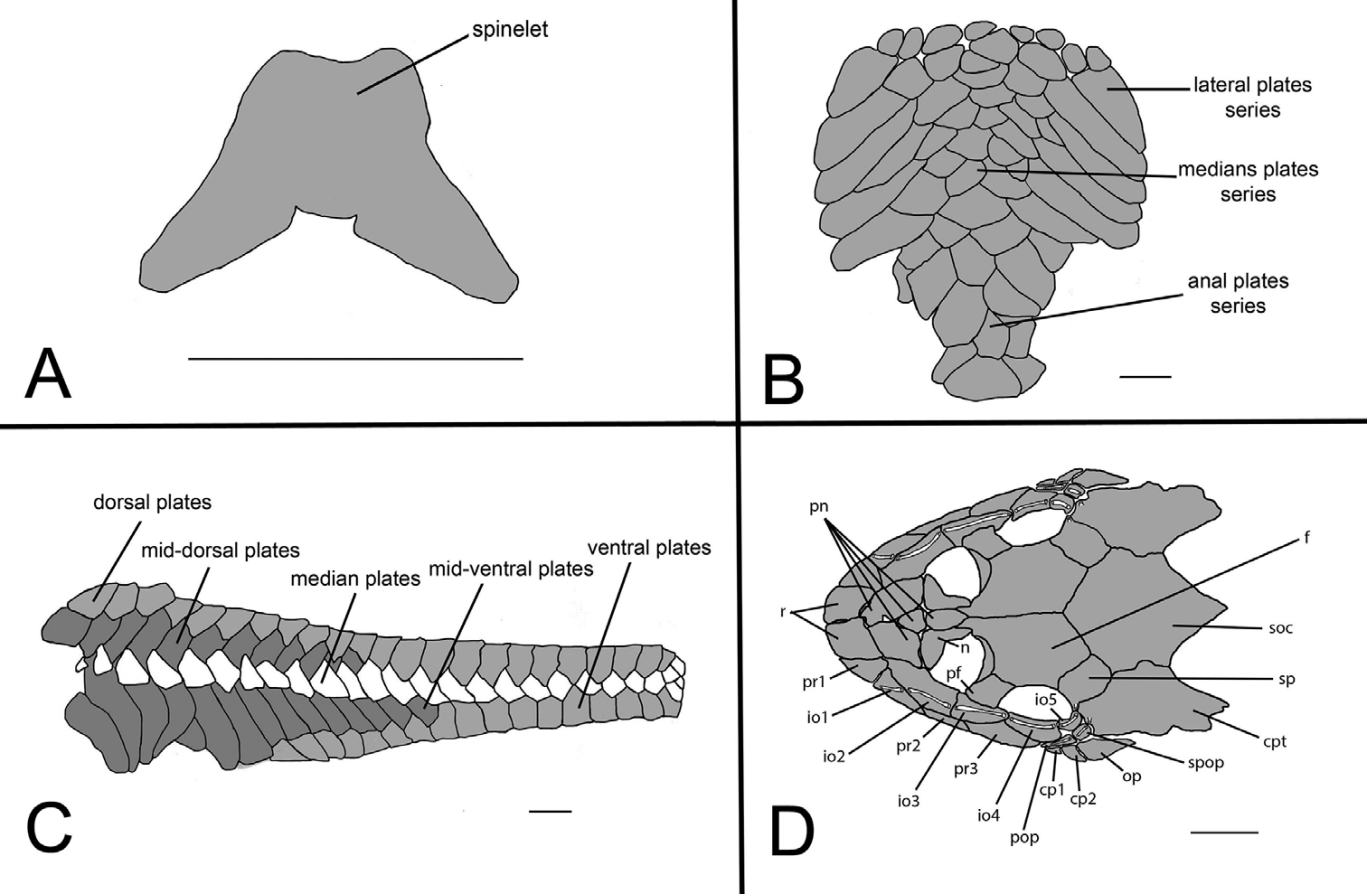
*Curculionichthys sagarana*, NUP 9715, 23.3 mm SL. **A** Spinelet shape **B** Ventral view of abdominal plates **C** Lateral trunk plates **D** Cranial bones plates of the head in dorsal view. Scale bar: 0.5 mm (**A**); 1 mm (**B, C, D**).

Dorsal fin ii, 7; dorsal fin spinelet short and V-shaped (Fig. 12A); dorsal fin lock functional; dorsal fin origin slightly posterior to pelvic fin origin. Tip of adpressed dorsal fin reaching anal fin insertion. Pectoral fin i, 6; its tip reaching beyond pelvic fin insertion when depressed. Presence of pectoral axillary slit between pectoral fin insertion and lateral process of cleithrum variable; absent in some specimens. Pectoral spine supporting odontodes on ventral, anterior and dorsal surfaces. Pelvic fin i, 5; tip of pelvic fin unbranched ray almost reaching anal fin origin when depressed in females and reaching anal fin origin in males. Pelvic fin unbranched ray with dermal flap along dorsal surface in males. Anal fin i, 5; distal margin slightly convex. Caudal fin i, 7-7, i; slightly emarginate; both unbranched rays of same size. Adipose fin absent. Total vertebrae 28.

Body covered with bony plates, except above head, around pectoral and pelvic-fin origins and on dorsal fin base. Cleithrum and coracoid entirely exposed. Arrector fossae partially to completely enclosed by ventral lamina of coracoids. Abdomen entirely covered by plates (Fig. 12B); lateral plates series with elongate and large plates formed by two lateral plate series, similar in size; median plates formed by two to three irregular plate series reaching anal shield and lateral plate series; anal plates series covered by large square plates. Body entirely covered laterally by plates (Fig. 12C); mid-dorsal plates poorly developed and reaching end of dorsal fin base; median plates series continuous in median portion of body; mid-ventral plates reaching caudal peduncle origin. Dorsal portion of caudal peduncle with one unpaired platelet.

Parts of dorsal head bone plates presented in Fig. 12D. Snout tip formed by one pair of rostral rectangular-shaped plates (r). Nasal (n) almost rectangular forming anterior medial nostril margin in contact posteriorly with frontals (f) and anteriorly and laterally with pre-nasals (pn). Pre-nasals (pn) positioned posteriorly of rostral plates (r), formed by two large and one small triangular-shaped plates, and one elongate oval shaped between nares. Top of head composed by compound pterotic (cpt), parieto supraoccipital (soc) and frontal (f), largest bones of head, and prefrontal (pf) and sphenotic (sp). Compound pterotic (cpt) fenestrated randomly distributed. Posterior rostrum plates pr1-pr2 small and triangular-shaped; pr4-pr3 largest, and rectangular-shaped. Infraorbital plate series complete (io1-io5), present just above posterior rostrum series, all covered by latero-sensory canal system; io2 largest and io5 smallest; io3, io4 and io5 forming inferior orbital margin of eyes; preopercle (pop) elongated and rectangular, covered by latero-sensory canal; preopercle present under io4, and upper cp1, cp2 and op. Supra-opercular plate (spop) present just above preopercle, covered by latero-sensory canal. Subocular cheek plates (cp1-cp2) and operculum (op) form posterior lateral margin of head.

**Color in alcohol.** Ground color of dorsal and ventral region of head and trunk pale yellowish; dorsal portion darker than ventral. Four dark saddles along dorsal portion of body: first at dorsal fin origin; second at end of dorsal fin; third at middle of caudal peduncle; and fourth at upper caudal peduncle adpressed ray origin. Dorsal, pectoral, and pelvic fins hyaline. Caudal fin hyaline, with dark blotch limited to caudal peduncle base, and with dark chromatophores irregular distributed almost forming one band.

**Sexual dimorphism.** Adults males have a papilla in urogenital opening (*vs.* absent in females); a longer pelvic fin that extends beyond anal fin origin (*vs.* pelvic fin not reaching anal fin origin in females); nares opening wider (*vs.* nares opening narrower); and an unbranched pelvic fin ray supporting a large dermal flap along its dorsal surface. Both sex have a membrane on anal opening; however, this membrane is more developed in females than in males, covering almost the entire urogenital opening (see reference to this last character in Roxo et al. 2014b).

##### Distribution.

The new species *Curculionichthys sagarana* are known from two localities along Rio das Velhas drainage: one at Rio 13 de Maio, one at Pardo Grande, and one at Rio Curimataí, all in Rio São Francisco basin, Minas Gerais State, Brazil (Fig. 8).

##### Etymology.

The specific name “sagarana” is a hybrid of two words, “saga” of Germanic origin that means heroic song and “rana” from Tupi-Guarani language that means “similarity”. The word sagarana is in reference to the book of a Brazilian author João Guimarães Rosa published in 1946 about the history of people from Minas Gerais State living in the region of Rio das Velhas.

##### Comparative remarks.

The new species *Curculionichthys sagarana* is similar in external morphology with *Curculionichthys insperatus*, primarily the general pattern of coloration of the body. However, *Curculionichthys sagarana* can be distinguished by the presence of one unpaired platelet on the dorsal portion of caudal peduncle, a character apparently present only in this new species, more premaxillary and dentary teeth, and small, inconspicuous odontodes forming rows on the head and trunk.

### Key to species of *Curculionichthys*

**Table:** 

1	Odontodes forming longitudinally aligned rows (one odontode after the other, but not necessarily forming parallel series) on head (more prominent) and trunk	**2**
−	Odontodes not forming longitudinally aligned rows on head and trunk (Rio Paranaíba basin)	***Curculionichthys piracanjuba***
2	Cleithrum with an area free of odontodes	**3**
−	Cleithrum completely covered with odontodes	**5**
3	Presence of contrasting dark geometric spots on the anterodorsal region of the body (Rio Paraguai basin)	***Curculionichthys paresi***
−	Absence of geometric spots on the anterodorsal region of the body	**4**
4	Presence of several dark-brown spots distributed on the body; the anterior profile of the head pointed; presence of 28 vertebrae (Rio Xingu basin)	***Curculionichthys sabaji***
−	Lacking of several dark-brown spots distributed on the body; the anterior profile of the head rounded; presence of 29 to 30 vertebrae (Rio Cuiabá basin)	***Curculionichthys coxipone***
5	Presence of one unpaired platelet on dorsal portion of caudal peduncle (Rio das Velhas basin)	***Curculionichthys sagarana***
−	Dorsal portion of caudal peduncle without unpaired platelets	**6**
6	Caudal fin hyaline, with dark blotch limited to caudal peduncle base; six to nine lateral abdomen plates	**7**
−	Caudal fin hyaline, with one dark strip extending from caudal peduncle base to the median caudal fin rays; four to five lateral abdomen plates (Rio Ivaí basin)	***Curculionichthys oliveirai***
7	Small and inconspicuous odontodes forming rows on the head and trunk; caudal fin hyaline, with one dark stripe extending from caudal peduncle base to the median caudal-fin rays, and for irregularly distributed dark chromatophores almost forming one band (Rio Tapajós basin)	***Curculionichthys luteofrenatus***
−	Conspicuous odontodes forming rows on the head and the trunk; caudal fin hyaline, with dark blotch limited to caudal peduncle base (Rio Paranapanema, Tietê and Grande basins)	***Curculionichthys insperatus***

### Comparative material

All from Brazil, except when stated otherwise:

*Corumbataia cuestae* Britski, 1997: LBP 3688, 3, 28.5−29.9 mm SL; Rio Araquá, municipality of Botucatu, São Paulo State.

*Curculionichthys insperatus* (Britski & Garavello, 2003): LBP 4945, 7, 27.3−29.9 mm SL, Rio Araquá, municipality of Botucatu, São Paulo State; LBP 6770, 8, 20.0−28.2 mm SL, ribeirão Cubatão, municipality of Marapoama, São Paulo State; LBP 13336, 1 female c&s, 26.0 mm SL, Rio Capivara, municipality of Botucatu, São Paulo State; LBP 13337, 2 females c&s, 27.4−28.6 mm SL, Rio Araquá, municipality of Botucatu, São Paulo State; MZUSP 22826, 1, 25.4 mm SL, paratype, Córrego Água Tirada, municipality of Três Lagoas, Minas Gerais State; MZUSP 24832, 1, 23.8 mm SL, paratype, Rio Corumbataí, municipality of Corumbataí, São Paulo State; MZUSP 78957, 29.6 mm SL, holotype, Rio Capivara, municipality of Botucatu, São Paulo State; MZUSP 78960, 31, 12.6−26.0 mm SL, paratypes, 5 c&s, 22.7−24.7 mm SL, Rio Pardo, municipality of Botucatu, São Paulo State; MZUSP 78965, 10, 15.6−28.6 mm SL, paratypes, 3 c&s, not measured, Rio Araquá, municipality of Botucatu, São Paulo State; MZUSP 78968, 5, 24.1−27.3 mm SL, paratypes, Córrego da Figueira, municipality of Lins, São Paulo State.

*Curculionichthys luteofrenatus* (Britski & Garavello, 2007): MZUSP 62593, 28.6 mm SL, holotype, Córrego Loanda, municipality of Cláudia, Mato Grosso State; MZUSP 62594, 8, 22.4−30.5 mm SL, paratypes, riacho Selma, municipality of Sinop, Mato Grosso State; MZUSP 87144, 8, 16.8−27.9 mm SL, paratypes, Córrego Loanda, municipality of Cláudia, Mato Grosso State.

*Curculionichthys oliveirai* (Roxo, Zawadzki & Troy, 2014b): MZUSP 115061, 26.4 mm SL, holotype, ribeirão Cambira, municipality of Cambira, Paraná State; LBP 13332, 1 male, 23.2 mm SL, 1 unsexed c&s, 23.7 mm SL, paratype, Rio Mourão, municipality of Campo Mourão, Paraná State; LBP 17578, 5, 25.4−30.4 mm SL, paratypes, Rio Mourão, between municipality of Engenheiro Beltrão and Quinta do Sol, Paraná State; NUP 3578, 15, 24.7−28.1 mm SL, 2 c&s, 25.5−27.6 mm SL,, paratypes, ribeirão Salto Grande, municipality of Maria Helena, Paraná State.

*Curculionichthys paresi* (Roxo, Zawadzki & Troy, 2014b): MZUSP 115062, 26.2 mm SL, holotype, riacho Águas Claras, municipality of Santo Afonso, Mato Grosso State; LBP 13351, 9, 14.7−24.3 mm SL, paratype, riacho Águas Claras, municipality of Santo Afonso, Mato Grosso State; LBP 13352, 1, 23.7 mm SL, paratype, riacho Águas Claras, municipality of Santo Afonso, Mato Grosso State; NUP 10928, 2 males, 23.2−24.2 mm SL, paratype, 2 c&s, 23.6−24.2 mm SL, riacho Águas Claras, municipality of Santo Afonso, Mato Grosso State.

*Curculionichthys piracanjuba* (Martins & Langeani, 2012): LBP 17256, 9, 17.2−26.3 mm SL, 1, c&s 27.1 mm SL, córrego sem nome, municipality of Morrinhos, Goiás State; NUP 5059, 1, 24.7 mm SL, Córrego Posse, municipality of Anápolis, Goiás State; MZUSP 110491, 3, 17.5−24.4 mm SL, paratypes, Rio Quente, municipality of Marcelãnia, Goiás State; NUP 10979, 3, 21.4−21.8 mm SL, ribeirão Bocaina, municipality of Piracanjuba, Goiás State.

*Curculionichthys* sp. 1: LBP 17531, 3, 23.3−25.8 mm SL, Rio Russo I, municipality of Tangará da Serra, Mato Grosso State.

*Curculionichthys* sp. 2: LBP 17485, 7, 19.0−24.1 mm SL, Igarapé Imambuaí, municipality of Itaituba, Pará State.

*Curculionichthys* sp. 3: LBP 1856, 2, 21.0−23.2 mm SL, Rio Insula, Barra do Garça, Mato Grosso State.

*Curculionichthys* sp. 4: MZUSP 87452, 3, 22.4−24.5 mm SL, unknown river, municipality of Streito, Maranhão State; MZUSP 87553: 3, 21.7−24.3 mm SL, unknown river, municipality of Feira Nova do Maranhão, Maranhão State.

*Hisonotus acuen* Silva, Roxo & Oliveira, 2014: MZUSP 115350, 25.9 mm SL, holotype, affluent of Rio Toguro, municipality of Querência, Mato Grosso State; LBP 15755, 16, 19.5−26.0 mm SL, paratypes, affluent of Rio Suiá-Missu, municipality of Ribeirão Cascalheira, Mato Grosso State; LBP 16274, 27, 20.2–29.1 mm SL, 2 c&s 23.6−24.2 mm SL, paratypes, affluent of Rio Culuene, municipality of Gaúcha do Norte, Mato Grosso State; LBP 16275, 29, 16.7−25.2 mm SL, 2 c&s 19.3−20.8 mm SL, paratypes, affluent of Rio Feio, municipality of Querência, Mato Grosso State; LBP 16278, 12, 18.8–25.1 mm SL, 2 c&s 26.8−27.1 mm SL, paratypes, Córrego Xavante, municipality of Primavera do Leste, Mato Grosso State.

*Hisonotus aky* (Azpelicueta, Casciotta, Almirón & Koerber, 2004): MHNG 2643.039, 2, 33.1−34.2 mm SL, paratypes, arroio Fortaleza, Argentina.

*Hisonotus armatus* Carvalho, Lehmann, Pereira & Reis, 2008: MZUSP 93884, 5, 37.6–44.4 mm SL, paratypes, arroio Arambaré, municipality of Pedro Osório, Rio Grande do Sul State.

*Hisonotus bocaiuva* Roxo, Silva, Oliveira & Zawadzki, 2013: MZUSP 112204, 24.2 mm SL, holotype, Córrego Cachoeira, municipality of Bocaiúva, Minas Gerais State; LBP 9817, 9, 3 c&s, 18.3−23.2 mm SL, paratypes, Córrego Cachoeira, municipality of Bocaiúva, Minas Gerais State.

*Hisonotus brunneus* Carvalho & Reis, 2011: MZUSP 104947, 4, 37.2−41.3 mm SL, paratypes, Rio Passo Novo, municipality of Cruz Alta, Rio Grande do Sul State.

*Hisonotus carreiro* Carvalho & Reis, 2011: MCP 40943, 3, 33.6−35.8 mm SL, arroio Guabiju, municipality of Guabiju, Rio Grande do Sul State.

*Hisonotus charrua* Almirón, Azpelicueta, Casciotta & Litz, 2006: LBP 4861, 1, 35.9 mm SL, arroio Guaviyú, Artigas, Uruguay; MHNG 2650.051, 1, 34.2 mm SL, paratype, arroio Aspinillar, Uruguay.

*Hisonotus chromodontus* Britski & Garavello, 2007: LBP 7964, 25, 24.0−28.3 mm SL, 4 c&s, 24.9−28.9 mm SL, Rio dos Patos, municipality of Nova Mutum, Mato Grosso State; LBP 7974, 26, 17.7–24.8 mm SL, Rio dos Patos, municipality of Nova Mutum, Mato Grosso State; LBP 12278, 2, 26.7−28.7 mm SL, 1 c&s, 26.7 mm SL, Rio Sumidouro, municipality of Tangará da Serra, Mato Grosso; MZUSP 45355, 25.9 mm SL, holotype, affluent of Rio Preto, municipality of Diamantino, Mato Grosso State; MZUSP 70758, 7, 19.4−23.9 mm SL, paratype, riacho Loanda, municipality of Sinop, Mato Grosso State; NUP 10924, 24, 19.5−31.5 mm SL, Rio Preto, municipality of Diamantino, Minas Gerais State.

*Hisonotus depressicauda* (Miranda Ribeiro, 1918): MZUSP 5383, 24.4 mm SL, paralectotype, municipality of Sorocaba, São Paulo State; LBP 17474, 5 c&s, 18.1−24.0 mm SL, Rio Araquá, municipality of Botucatu, São Paulo State.

*Hisonotus francirochai* (Ihering, 1928): LBP 13923, 22, 25.7−35.7 SL, córrego sem nome, municipality of Capitinga, Minas Gerais State; MZUSP 3258, 29.4 mm SL, lectotype, Rio Grande, São Paulo State.

*Hisonotus heterogaster* Carvalho & Reis, 2011: LBP 3335, 39, 20.8−30.1 mm SL, arroio sem nome, municipality of Rio Grande, Rio Grande do Sul State; MZUSP 104948, 3, 40.3−43.0 mm SL, paratypes, arroio Felício, municipality of Júlio de Castilho, Rio Grande do Sul State.

*Hisonotus iota* Carvalho & Reis, 2009: LBP 13072, 5, 32.3−33.0 mm SL, Rio Chapecó, municipality of Coronel Freitas, Santa Catarina State.

*Hisonotus laevior* Cope, 1894: LBP 3377, 1, 25.2 mm SL, arroio dos Corrientes, municipality of Pelotas, Rio Grande do Sul State; LBP 6037, 8, 33.4−47.0 mm SL, Rio Maquiné, municipality of Osório, Rio Grande do Sul State; LBP 13187, 7, 19.4−45.8 mm SL, córrego sem nome, municipality of Camaquá, Rio Grande do Sul State.

*Hisonotus leucofrenatus* (Miranda Ribeiro, 1908): LBP 2085, 7, 38.3−50.6 mm SL, Rio Sagrado, municipality of Morretes, Paraná State; LBP 6837, 36, 35.1−43.5 mm SL, Rio Fau, municipality of Miracatu, São Paulo State.

*Hisonotus leucophrys* Carvalho & Reis, 2009: LBP 13065, 6, 17.2−33.6 mm SL, Rio Ariranhas, municipality of Xavantina, Santa Catarina State; LBP 13073, 1, 36.8 mm SL, Rio Guarita, municipality of Palmitinho, Rio Grande do Sul State.

*Hisonotus megaloplax* Carvalho & Reis, 2009: LBP 13108, 6, 36.4−37.8 mm SL, córrego sem nome, municipality of Saldanha Marinho, Rio Grande do Sul State.

*Hisonotus montanus* Carvalho & Reis, 2009: LBP 13051, 3, 26.4−27.2 mm SL, Rio Goiabeiras, Vargem, SC; LBP 13055, 5, 24.8−31.9 mm SL, Rio Canoas, municipality of Vargem, Santa Catarina State.

*Hisonotus nigricauda* (Boulenger, 1891): LBP579, 16, 34.1−40.1 mm SL, Rio Guaíba, municipality of Eldorado do Sul, Rio Grande do Sul State.

*Hisonotus notatus* Eigenmann & Eigenmann, 1889: LBP 3472, 20, 21.0−34.3 mm SL, 3 c&s 25.0−26.5 mm SL, Rio Aduelas, municipality of Macaé, Rio de Janeiro; LBP 10742, 25, 24.4−43.3 mm SL, Rio Macabu, municipality of Conceição de Macabu, Rio de Janeiro State.

*Hisonotus notopagos* Carvalho & Reis, 2011: MZUSP 104943, 4, 35.3−37.3 mm SL, arroio Boici, municipality of Pinheiro Machado, Rio Grande do Sul State.

*Hisonotus prata* Carvalho & Reis, 2011: MCP 40492, 18, 19.5−33.2 mm SL, Rio da Prata, municipality of Nova Prata, Rio Grande do Sul State; LBP 9918, 14, 21.7−32.6 mm SL, Laguna dos Patos system, municipality of Nova Prata, Rio Grande do Sul State.

*Hisonotus ringueleti* Aquino, Schaefer & Miquelarena, 2001: FMNH 108806, 2, 25.7−32.2 mm SL, Rio Quaraí basin, Uruguay; LBP 13148, 1, 24.5 mm SL, arroio Putiá, municipality of Uruguaiana, Rio Grande do Sul State.

*Hisonotus vespuccii* Roxo, Silva & Oliveira, 2015a: MZUSP 115274, 32.6 mm SL, holotype, Rio São Francisco, municipality of Pirapora, Minas Gerais State; LBP 10421,18, 23.6–30.3 mm SL, 5 c&s sex not determined 20.2–29.6 mm SL, Rio São Francisco, municipality of Pirapora, Minas Gerais State.

*Hisonotus vireo* Carvalho & Reis, 2011: MZUSP 104946, 4, 30.4−39.5 mm SL, Rio dos Sinos, municipality of Caraá, Rio Grande do Sul State.

*Microlepidogaster arachas* Martins, Calegari & Langeani, 2013: LBP 10882, 3, 22.8−35.3 mm SL, Rio Paraná basin, municipality of Araxás, Minas Gerais State;

*Microlepidogaster dimorpha* Martins & Langeani, 2011: LBP 10683, 2, 28.8−35.6 mm SL, Rio Uberaba, municipality of Uberaba, Minas Gerais State.

*Otothyropsis marapoama* Ribeiro, Carvalho & Melo, 2005: LBP 4698, 6, 23.9−36.3 mm SL, ribeirão Cubatão, municipality of Marapoama, São Paulo State.

*Parotocinclus maculicauda* (Steindachner, 1877): LBP 2869, 15, 20.2−44.7 mm SL, Rio Fau, municipality of Miracatu, São Paulo State;

*Parotocinclus prata* Ribeiro, Melo & Pereira, 2002: LIRP 1136, 38, 19.8−41.9 mm SL, paratypes, ribeirão Quiricó, municipality of Presidente Olegário, Minas Gerais State.

*Parotocinclus robustus* Lehmann & Reis, 2012: LBP 8258, 29, 18.7−39.1 mm SL, Córrego Cachoeira, municipality of Bocaiúva, Minas Gerais State.

*Pseudotothyris obtusa* (Miranda Ribeiro, 1911): LBP 6822, 70, 22.5−31.7 mm SL; tributary of Rio Preto, municipality of Itanhaém, São Paulo State.

*Rhinolekos britskii* Martins & Langeani, 2011: LBP 7253, 21.9−34.7 mm SL; tributary of Rio Paranaíba, municipality of Pires do Rio, Goiás State.

*Rhinolekos capetinga* Roxo, Ochoa, Silva & Oliveira, 2015b: MZUSP 116102, holotype, 37.5 mm SL, Córrego da Branca, municipality of Água Fria de Goiás, Goiás State; LBP 19001, paratypes (35, 26.8–39.5 mm SL, 3 c&s, 37.2–32.6 mm SL, 9 sex not determined and not measured), Córrego da Branca, municipality of Água Fria de Goiás, Goiás State.

*Schizolecis guntheri* (Miranda Ribeiro, 1918): LBP 2123, 21, 28.4−36.3 mm SL, Rio Parati-Mirim, municipality of Parati, Rio de Janeiro State; LBP 3546, 77, 20.9−35.8 mm SL, coastal drainage, municipality of Ubatuba, São Paulo State.

## Supplementary Material

XML Treatment for Curculionichthys

XML Treatment for Curculionichthys sabaji

XML Treatment for Curculionichthys coxipone

XML Treatment for Curculionichthys sagarana
